# Synthesis and Optimization of Highly Bright Silver-Coated Au Nanostars with Tunable Plasmonic Properties

**DOI:** 10.1021/acsnanoscienceau.5c00075

**Published:** 2025-09-11

**Authors:** Judith Peñas-Farré, Xiaofei Xiao, Vincenzo Giannini, Xavier Mateos, Luca Guerrini, Nicolas Pazos-Perez

**Affiliations:** 1 Department of Physical and Inorganic Chemistry. 16777 Universitat Rovira i Virgili, Carrer de Marcel·lí Domingo 1, Tarragona 43007, Spain; 2 559361 Technology Innovation Institute, Building B04C, Masdar City, Abu Dhabi P.O. Box 9639, United Arab Emirates; 3 Instituto de Estructura de la Materia (IEM-CSIC), Consejo Superior de Investigaciones Científicas, Serrano 121, Madrid 28006, Spain; 4 Centre of Excellence ENSEMBLE3 sp. z o.o., Wolczynska 133, Warsaw 01-919, Poland

**Keywords:** nanostars, plasmonic, core−shell, bimetallic, surface-enhanced Raman scattering, Ag overgrowth

## Abstract

Silver-coated gold nanostars (AuNSt@Ag) offer a powerful platform for plasmon-enhanced sensing, yet their fabrication often compromises structural sharpness and spectral tunability. Here, we report a robust and flexible method for synthesizing AuNSt@Ag with precisely controlled localized surface plasmon resonance (LSPR) across a broad spectral range, achieved by systematically optimizing multiple synthetic parameters. Strikingly, surface-enhanced Raman scattering (SERS) performance reached a maximum for bimetallic nanostars with LSPR maxima near 605–615 nm, regardless of excitation wavelength (633 or 785 nm). This reveals that local near-field enhancement at Ag-coated tips, rather than spectral overlap, governs SERS efficiency in these AuNSt@Ag systems. The optimized AuNSt@Ag structures outperform previously reported analogues, exhibiting significantly enhanced SERS capabilities, including an 80-fold increase in signal compared to optimized monometallic AuNSt resonant with the 785 nm laser line. These findings establish a new design paradigm for highly tunable and high-performance plasmonic substrates for analytical sensing applications.

## Graphical abstract

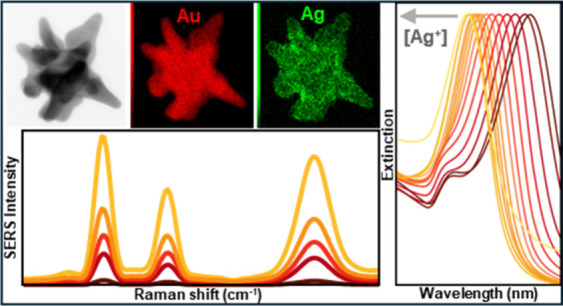

## Introduction

Over the past two decades, the field of plasmonic nanostructures has witnessed remarkable advancements, transitioning from a field of immense theoretical potential to one driving major technological innovations. Most notably, gold (AuNPs) and silver nanoparticles (AgNPs) have garnered significant attention due to their unique optical and electronic properties.^1,2^ One of their most intriguing characteristics is the localized surface plasmon resonance (LSPR) effect, which is highly dependent on particle composition, size and shape. Such dependency enables precise tuning of their optical properties, thereby enhancing their effectiveness in sensing, imaging, catalysis, and therapeutic applications.^2,3^ Silver-based nanoparticles typically outperform gold ones in key plasmonic properties, such as higher extinction cross sections, larger resonance quality factors, and lower optical losses. However, their lower chemical resistance limits practical applications where stability and longevity are essential.^4,5^ As a result, selecting a metal for plasmonic devices often involves balancing silver superior optical properties with gold exceptional chemical stability and greater flexibility in tuning particle morphologies.^6^

Regarding particle morphology, researchers have emphasized the superior optical properties of anisotropic nanomaterials compared to their isotropic counterparts.^7,8^ Among these, star-shaped colloids stand out due to their unique optical and colloidal features, making them highly attractive for various applications.^9^ Indeed, gold nanostars (AuNSt) exhibit distinctive plasmon hybridization modes resulting from core-tip interactions.^10^ The primary plasmonic activity of AuNSt originates from their sharp tips, which concentrate extremely high electromagnetic (EM) field enhancements,^9–11^ while the core plays a crucial role in enhancing the excitation cross-section and the localized EM field at the tips. This makes AuNSt an elite platform for photothermal applications and for plasmonic-enhanced optical effects, such as SERS.^9^ Furthermore, the ability to tailor their morphology through controlled synthesis parameters enhances the attractiveness of these nanomaterials, as it allows fine modulation of their LSPR position from the visible to the NIR range.^9,11,12^ Precisely tuning plasmonic resonances in metallic nanoparticles is essential for maximizing their optical response and optimizing the design of advanced systems.^3^

On the other hand, composite anisotropic nanomaterials are particularly appealing due to their synergistic effects, which enhance their overall properties. In particular, bimetallic Au/Ag nanomaterials have been shown to exhibit superior catalytic, electronic, and optical performance compared to their monometallic counterparts.^13,14^ Most notably, gold core/silver shell (Au@Ag) nanoparticles displayed greater SERS efficiency than pure gold or silver nanoparticles,^15^ while the electron compensation effect from Au to Ag increases electron density on the Ag shell, reducing its tendency to oxidize.^16,17^ This is crucial for enhancing the long-term stability of these hybrid nanomaterials in complex media.^16^

In such Au@Ag structures, the thickness of the silver shell plays a crucial role in determining the overall features of the composite material. Thus, controlled variation of the thickness of the Ag coating facilitates the precise tuning of the LSPR band.^18^ On the other hand, it significantly affects both plasmonic enhancement and overall chemical stability.^18–20^ For instance, a recent study by Feng et al.^16^ identified an optimal Ag shell thickness of ∼ 2.4 nm for maximizing charge transfer effects and biocompatibility in both *in vitro* and *in vivo* applications. It is important to stress that, besides the Au core morphology, the final shape of Au@Ag particles depends on several other factors, such as the gold crystal facets, the choice of Ag precursor, the selection of reducing and capping agents, solvent polarity, and temperature.^21^

To further enhance the plasmonic properties of monometallic AuNSt, researchers have explored silver coating strategies. One of the earliest reports described the deposition of a silver layer on AuNSt using hydrogen peroxide (H_2_O_2_) as a reducing agent in the presence of a strong base (NH_3_).^22^ However, reproducing this method has proven challenging. A more robust and widely adopted approach was later introduced by Vo-Dinh and colleagues, who developed a two-step seed-mediated growth method for coating gold nanostars with silver (AuNSt@Ag).^18^ Since then, this methodoloy has undergone several refinements,^23–33^ leading to significant advancements in the ultrasensitive detection of environmentally and biologically relevant analytes.^25,26,28,31,34–36^ In this well-established approach, surfactant-free AuNSt are dispersed in a silver ion solution, where a mild reducing agent promotes the controlled reduction of Ag^+^ directly onto the gold surface, effectively preventing bulk nucleation. Ascorbic acid (AA) is the preferred mild reducing agent for Ag^+^, offering controlled kinetics, low toxicity, biocompatibility, and precise control over silver shell thickness.^34^ The process is typically carried out without surface-directing agents in the growth medium. Similarly, monometallic AuNSt are usually synthesized via a surfactant-free method for use as seeds in subsequent silver deposition.^23–26,28,37^ Nonetheless, AuNSt stabilized with surface ligands such as triton-X ^30,31^ and CTAB ^32,33^ have been also reported for this purpose. However, in such protocols, silver deposition typically occurs nonhomogeneously on the AuNSt surfaces: first, a spherical Ag layer is formed at the central core of the AuNSt and, only after significant expansion of this inner shell and major reshaping of the original NSt morphology, Ag finally coats the gold tips.^19,28,30,32,33,35,37–39^ Both theoretical models and experimental findings show that the greatest EM enhancement in this class of AuNSt@Ag occurs when the silver shell predominantly surrounds the AuNSt core, with the gold surface of the tips remaining exposed.^18,36^ Such an intensification of local electric fields commonly boosted the SERS performance by a factor of approximately 5 to 10.^18,30,31,36,37,40,41^ On the other hand, it has been also reported that the partial coating of the Ag layer led to significant variations in signal intensity across the substrate, minimizing the electrical coupling between Au and Ag at the tips of the bimetallic nanostars.^40^

Few studies suggest that slight modifications to the established methodology can enhance the uniformity of silver deposition, potentially directing more Ag toward the gold tip and reducing inconsistencies in layer thickness.^23,25^ Very recently, Zhang et al.^40^ introduced a novel strategy using polyethylenimine (PEI) to control silver ion deposition. The polymer forms a PEI-Ag^+^ complex in solution, which selectively binds to gold surfaces with varying curvatures, influencing both deposition rate and uniformity. As a result, this process directs silver growth outward from the core along the branches, eventually achieving complete tip coverage.

Nonetheless, to our knowledge, current methodologies do not offer precise and reproducible control over the optical properties of gold nanostars during silver deposition, particularly in terms of finely tuning their LSPR. This limitation is also expected to hinder the maximization of SERS performance in AuNSt@Ag nanostructures. In this study, we establish and systematically refine a synthetic strategy that enables homogeneous silver deposition from the early stages of growth, while effectively preventing bulk silver nucleation even at higher silver concentrations. This approach allows for reliable modulation of the LSPR across the visible–near-infrared range, while preserving the nanostar morphology. Most notably, the resulting bimetallic colloids exhibit unprecedented SERS enhancements compared to their monometallic AuNSt counterparts under different laser excitations, including an 80-fold greater response relative to optimized AuNSt with a tip-plasmon resonance matched to the 785 nm laser line. Overall, the proposed methodology provides deeper insight into the design and optimization of AuNSt@Ag substrates and offers a versatile, scalable, and highly effective platform for the development of advanced plasmonic sensing systems.

## Experimental Section

### Materials

PVP (MW = 40000) was purchased from MP biomedicals. Gold­(III) chloride trihydrate (99.9%, HAuCl_4_·3H_2_O), silver nitrate (≥99.0%, AgNO_3_), trisodium citrate dihydrate (≥99.5%, C_6_H_5_Na_3_O_7_·2H_2_O), l-ascorbic acid (99%, AA), tween 20, and absolute ethanol (≥99.9%, EtOH) were obtained from Sigma-Aldrich. DMF (≥99%) was acquired from Fluka. Thiophenol (99%, TP) and mercaptoundecanoic acid (99%, MUA) were obtained from Fisher Scientific. All reactants were used without further purification. Milli-Q water (18 MΩ cm^–1^) was used in all aqueous solutions, and all the glassware was cleaned with aqua regia before the experiments.

### Synthesis of Au Seeds

Spherical, multitwinned gold nanoparticles (Au NPs) of approximately 12 nm diameter were synthesized via a modified Turkevich method.^42,43^ Briefly, 150 mL of Milli-Q water was heated to boiling under reflux to prevent solvent evaporation. Once boiling commenced, 3.3 mL of sodium citrate (0.1 M) was added under vigorous stirring (1200 rpm), resulting in a final citrate concentration of 2.2 mM. After 10 min, an aqueous HAuCl_4_ solution (238.77 μL, 0.1 M) was introduced, and the reaction was maintained for 30 min under continuous boiling and stirring. During this time, the solution color transitioned from colorless to purple and finally to deep red, indicating nanoparticle formation. After 30 min, the reaction mixture was cooled to room temperature. The resulting Au seeds (∼12 nm, ∼ 2 × 10^12^ NPs/mL, [Au^0^] = 1.6 × 10^–4^ M) were stabilized by negatively charged citrate ions, ensuring good dispersion in water. Polyvinylpyrrolidone (PVP) was used as a phase transfer agent to transfer the particles into ethanol.^44^ Specifically, the gold colloid (150 mL) was added dropwise under vigorous stirring to a previously sonicated (30 min) aqueous PVP solution (150 mL, 0.27 mM). The mixture was stirred (600 rpm) at room temperature for 24 h to ensure complete PVP adsorption onto the Au NP surface. For ethanol transfer, the solution volume was first reduced by evaporation using a hot plate until approximately 140 mL remained. The Au seeds were then divided into four centrifuge tubes (35 mL each) and centrifuged (9000 rpm, 45 min). After discarding the supernatant, the particles were redispersed in ethanol, yielding a final [Au^0^] of 18.7 × 10^–4^ M, ∼ 2.11 × 10^13^ NPs/mL.

### Synthesis of AuNSt

AuNSt were prepared following a modification of the standard PVP/DMF approach.^12,45^ Poly­(vinylpyrrolidone) (7 g) was dissolved in 20 mL of N,N-dimethylformamide (DMF). After complete dissolution, additional DMF was added to reach a final volume of 35 mL, and the mixture was sonicated for 30 min. Subsequently, an aqueous solution of HAuCl_4_ (106.9 μL, 0.1019 M) was added, and the mixture was vigorously shaken. Immediately afterward, 260 μL of PVP-coated Au seeds ([Au] = 18.7 × 10^–4^ M) were rapidly introduced, followed by further shaking. Within 15 min, the solution turned blue, indicating the formation of AuNSt. The reaction mixture was left undisturbed for 24 h to ensure the complete reduction of all reactants. Multiple centrifugation steps were carried out to remove DMF and excess of PVP. The solution was divided into four 15 mL centrifuge tubes, each containing approximately 8.75 mL. To each tube, 6.25 mL of ethanol (EtOH) was added (resulting in approximately a 1:1 DMF/EtOH ratio). The first centrifugation step was performed at 7500 rpm for 40 min, followed by six additional centrifugation steps at 7000 rpm for 10 min each. In all steps except the last, the particles were resuspended in 35 mL of EtOH. In the final centrifugation step, the particles were resuspended in the required volume of EtOH to obtain a final Au^0^ concentration of 7.2 × 10^–4^ M (∼4 × 10^11^ NPs/mL). The synthesized AuNSt exhibited a maximum LSPR peak at 800 nm. Additionally, AuNSt with a maximum LSPR peak at 780 and 820 nm were also produced, with the same protocol, by adding 290 and 230 μL of the PVP-coated Au seeds, respectively.

### Optimized Synthesis of AuNSt@Ag

After seven washing steps, 180 μL of the purified AuNSt (final [Au^0^] = 7.2 × 10^–4^ M) were mixed with 1318 μL of ethanol and 500 μL of water to achieve an EtOH/H_2_O ratio of 3. Next, 6 μL of sodium citrate (0.1 M) were added, followed by varying amounts of AgNO_3_ (0.01 M) and ascorbic acid (AA, 0.01 M). The solution was vigorously mixed and left undisturbed for 24 h. After this time, the evolution of the Ag-coated AuNSt (AuNSt@Ag) was monitored by UV–Vis spectroscopy. The AgNO_3_/AA molar ratio was maintained at 1.67. Specifically, for AgNO_3_ (0.01 M) volumes of 10, 9, 8, 7, 6, 5, 4, 3, 2, and 1 μL, the corresponding AA (0.01 M) volumes used were 6, 5.4, 4.8, 4.2, 3.6, 3.0, 2.4, 1.8, 1.2, and 0.6 μL, respectively.

The resulting AuNSt@Ag samples were used directly in SERS experiments without further processing to ensure equal nanoparticle concentration. Alternatively, the colloids can be stabilized using Tween 20 to facilitate centrifugation and purification without nanoparticle aggregation. In the latter case, 78 μL of Tween 20 (0.1 M) was added to 2 mL of colloidal solution, followed by a 10 min incubation and centrifugation at 4000 rpm for 10 min. The purified Au@Ag nanostars can then be resuspended in Milli-Q water. Note that all SERS experiments were performed directly after the Ag growth without the addition of Tween 20.

### Optimization of AuNSt@Ag Synthesis

The synthesis of AuNSt@Ag was optimized through an iterative process, where individual parameters were systematically screened while keeping all other conditions constant. The key parameters evaluated included the [Ag^+^]/[AA] ratio, the EtOH/H_2_O ratio, and the influence of stabilizing agents such as sodium citrate and polyvinylpyrrolidone (PVP). Additionally, the effects of washing steps and the silver precursor concentration relative to the Au nanostar (AuNSt) seeds were studied. This systematic optimization allowed for the precise tuning of the AuNSt@Ag synthesis conditions, ensuring reproducibility and optimal plasmonic properties.

#### Effect of Reducing Agent: Variation in [Ag^+^]/[AA] Ratio

To determine the optimal [Ag^+^]/[AA] ratio, a series of syntheses were conducted with ratios ranging from 10 to 2, using an EtOH/H_2_O ratio of 1:1 and five washing steps on the AuNSt seeds.

#### Impact of EtOH/H_2_O Volume Ratio

The effect of solvent composition was studied by varying the EtOH/H_2_O volume ratio from 9 to 0.12. Additionally, an EtOH/H_2_O ratio of 183 was tested, representing the minimal water content possible derived solely from AgNO_3_, AA, and trisodium citrate solutions.

#### Influence of Stabilizing Agents: Sodium Citrate

To evaluate the role of sodium citrate as a stabilizing agent, we varied its concentration from 0 to 4.5 × 10^–4^ M.

#### Influence of Stabilizing Agents: Polyvinylpyrrolidone (PVP) Concentration

The effect of PVP concentration was investigated in the range of 0 to 4.5 × 10^–4^ M. The highest intensity was observed at a sodium citrate concentration of 3.5 × 10^–4^ M. However, since the best results were obtained when no PVP was added, additional tests were performed to optimize the number of washing steps. Ag growth was tested using AuNSt subjected to 5, 6, 7, 8, and 9 centrifugation cycles.

#### Re-evaluation of Reducing Agent: Variation in [Ag^+^]/[AA] Ratio

Since the EtOH/H_2_O ratio and residual PVP were modified during the optimization process, the [Ag^+^]/[AA] ratio was re-evaluated. A new series of tests was performed with concentration ratios ranging from 2.5 to 1.

#### Effect of [Ag^+^]/[Au^0^] Concentration Ratio

Two experimental approaches were employed to investigate the role of silver precursor concentration relative to the number of AuNSt seeds. In the first approach, the AuNSt concentration was kept constant ([Au^0^] = 6.6 × 10^–^^5^ M), while the silver precursor concentration was systematically varied from 0.5 × 10^–5^ M to 5.0 × 10^–5^ M in increments of 0.5 × 10^–5^ M, maintaining a fixed [Ag^+^]/[AA] ratio of 1.67. In the second approach, the Ag^+^ concentration was kept constant at 1 × 10^–5^ M, while the AuNSt seed concentration ([Au^0^]) was adjusted between 3 × 10^–5^ M and 7 × 10^–5^ M in increments of 1 × 10^–5^ M.

### Numerical Simulations

To investigate the linear optical response of the nanostars, the extinction spectra were computed using the finite-difference time-domain (FDTD) method implemented in Lumerical FDTD Solutions. The nanostar geometry comprised a central spherical core with six protruding tips of varying lengths and orientations, all embedded in a surrounding medium with a refractive index of n = 1.36. Each nanostar was modeled as a composite structure consisting of a gold nanostar core and a silver nanoshell, as illustrated in the schematic. In all simulations, an x-polarized plane wave was normally incident from the negative *z*-axis direction. To compute the extinction cross-section, the Total-Field Scattered-Field (TFSF) source and corresponding analysis toolset within Lumerical were employed. Perfectly matched layers (PMLs) were applied in all spatial directions to effectively absorb outgoing waves and minimize artificial reflections. A uniform mesh size of 0.75 nm was used to balance computational accuracy with memory and time constraints. The wavelength-dependent permittivity values for gold and silver were obtained from the CRC Handbook data, as provided within the Lumerical material library.^46^

### Surface Functionalization for SERS Characterization

Different aliquots of an ethanolic TP solution (10^–4^ M) were rapidly added under stirring to 1 mL of AuNSt@Ag dispersion ([Au^0^] = 6.6 × 10^–5^ M, [Ag^0^] = 4 × 10^–5^ M, λ_max_ = 640 nm), resulting in final TP concentrations ranging from 7 × 10^–8^ M to 1 × 10^–5^ M. Additional Milli-Q water was added when needed to equalize the final volumes in each sample. The mixtures were allowed to react for 2 h before surface-enhanced Raman spectroscopy (SERS) analysis. An identical procedure was applied to pure AuNSt ([Au^0^] = 6.6 × 10^–5^ M). In a subsequent approach aimed at preventing colloidal aggregation, the as-prepared AuNSt@Ag particles were prefunctionalized with 11-mercaptoundecanoic acid (MUA) to enhance colloidal stability.^47^ Specifically, 11.6 μL of a 10^–5^ M MUA ethanolic solution was rapidly added under vigorous stirring to 1 mL of the AuNSt@Ag dispersion, yielding a final MUA concentration of 1 × 10^–7^ M. Stirring was continued for 2 h to ensure complete surface functionalization. Subsequently, 11.6 μL of a 10^–4^ M TP stock solution in ethanol was added under vigorous stirring to achieve a final TP concentration of 1 × 10^–6^ M. Gentle stirring was continued for 2 h before SERS measurements. This two-step functionalization protocol was also applied to both monometallic AuNSt samples (AuNSt seeds with LSPR centered at ∼ 815 nm and AuNSt with LSPR centered at ∼ 780 nm) under identical measurement conditions and with the same nanoparticle concentration ([Au^0^] = 6.6 × 10^–5^ M). All quantitative SERS measurements were performed in triplicate (N = 3) on independently prepared aliquots.

### Optical, Morphological, and Compositional Characterization

Optical and morphological characterization of the nanomaterials were carried out by UV–vis spectroscopy (Agilent Varian CARY 5000) and Transmission Electron Microscope (JEOL 1011 operating at 100 kV), and High Resolution TEM (JEOL F200 operating at 200 kV with X-ray microanalysis). TEM samples were prepared by drying colloidal suspensions on carbon–Formvar-coated 200 mesh copper grids. SERS experiments were carried out on the colloidal suspension by using a Renishaw Raman inVia system equipped with two laser lines (633 and 785 nm) and 1200 I/mm grating. The laser was focused into the bulk of the colloidal suspension using a nonimmersion macrolens collector

## Results and Discussion

In Scheme 1, we present a general overview of the synthetic process used to obtain gold–silver core–shell nanostars (AuNSt@Ag). First, AuNSt were prepared following a modification of the standard N,N-dimethylformamide (DMF)-polyvinylpyrrolidone (PVP) method designed by Liz-Marzan’s group.^12^ In this seed-mediated growth process, nanostars are grown on PVP-coated spherical gold seeds (∼12 nm diameter), then purified by centrifugation, and finally resuspended in ethanol. This method is widely used due to its ability to produce homogeneous nanostars with tunable plasmonic properties.^9^ Subsequently, the AuNSt were further purified through several centrifugation cycles to remove most of the PVP from the gold surface and, finally, resuspended in ethanol. The silver coating was then carried out at room temperature and constant pH (∼ 6) by mixing an aliquot of AuNSt with an aqueous solution of ascorbic acid (AA) and silver nitrate. To ensure colloidal stability in the ethanol/water mixture, citrate was also included as the stabilizing agent. Indeed, citrate anions adsorb onto the nanoparticle surface providing electrostatic stabilization, while promoting biocompatibility and facilitating further surface functionalization. The so-formed colloidal samples were left undisturbed 24 h before being analyzed. We decided to limit the full synthesis process to 1 day to achieve an optimal balance between colloidal stability, synthesis reproducibility, morphological control and cost-effectiveness. The resulting AuNSt@Ag samples were used directly for further characterization. Alternatively, colloids can be stabilized using Tween 20 (Figure S1) to facilitate further centrifugation and purification without nanoparticle aggregation.^48^

**Scheme 1:**
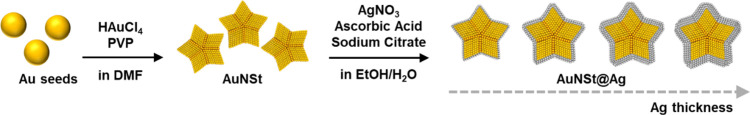
Outline of the Synthetic Process

### UV–vis Characterization

AuNSt exhibit a unique optical response characterized by two primary plasmonic contributions: (1) a band in the visible region corresponding to the dipolar mode of the core, and (2) a highly tunable tip-plasmon mode, whose position is strongly influenced by the length, sharpness, and density of the nanostar branches (Figure 1A).^9^ In this work, we selected the ∼ 600–800 nm range as the target interval for tuning the tip-plasmon resonance of AuNSt@Ag, as it includes the wavelengths of two commonly used laser lines in Raman spectrometers (633 and 785 nm). To this end, we first synthesized AuNSt with an LSPR maximum sufficiently red-shifted relative to the latter, in the 800–820 nm range (Figure 1A).

**Figure 1:**
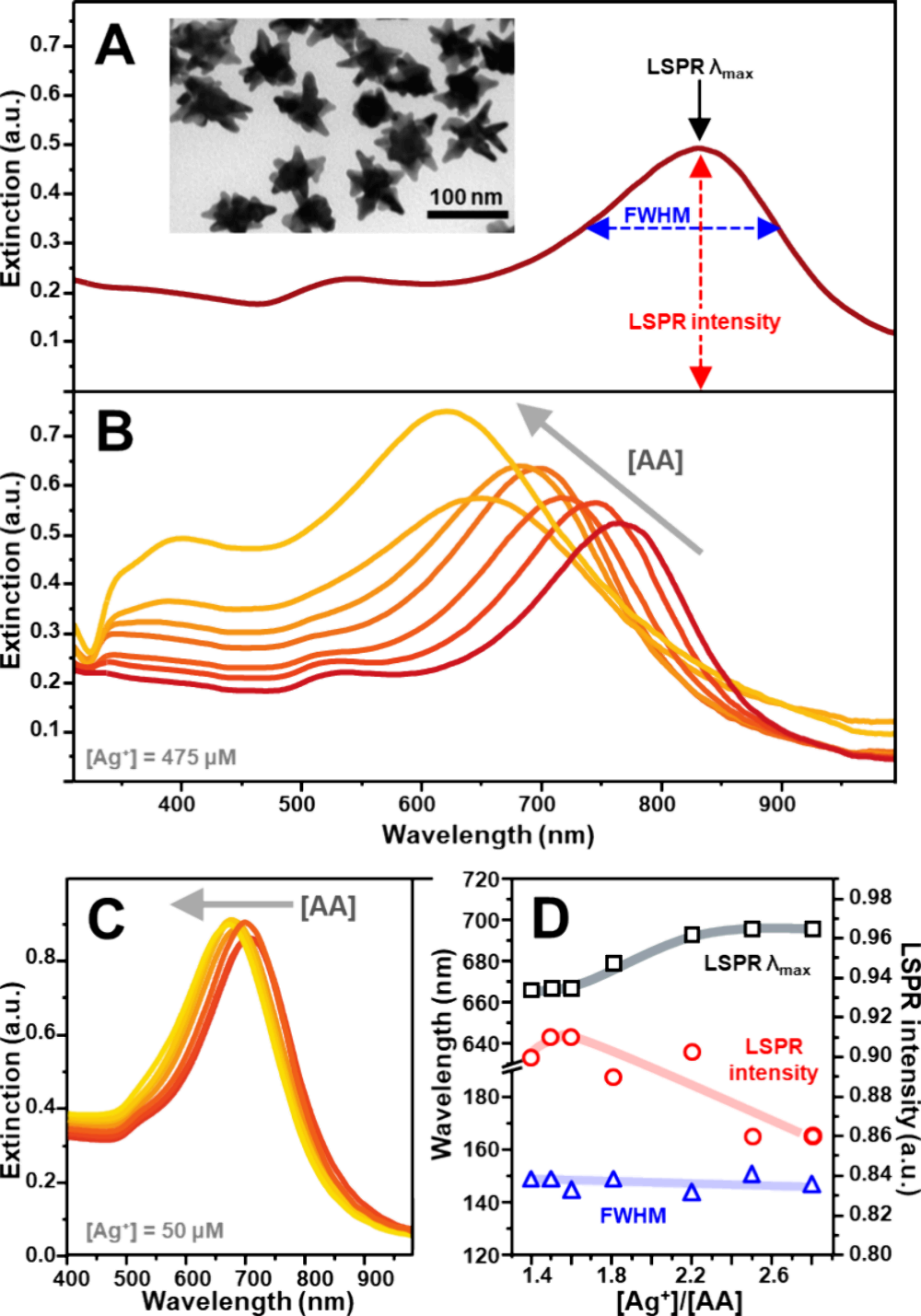
(A) Extinction spectrum of AuNSt along with their key morphological parameters and a representative TEM image (scale bar = 100 nm). (B) Extinction spectra of AuNSt@Ag obtained under the following growth conditions: [Au^0^] = 9.5 × 10^–5^ M, [Ag^+^] = 4.75 × 10^–4^ M, [citrate] = 2.5 × 10^–^^4^ M, with an EtOH/H_2_O volume ratio 1:1. The ascorbic acid (AA) concentration was varied to yield [Ag^+^]/[AA] ratios from 5 to 75. (C) Extinction spectra of AuNSt@Ag obtained under the following growth conditions: [Au^0^] = 9.5 × 10^–5^ M, [Ag^+^] = 5 × 10^–5^ M, [citrate] = 2.5 × 10^–4^ M, with an EtOH/H_2_O volume ratio 1:1. The [Ag^+^]/[ AA] ratio was varied from 1.4 to 2.8. (D) The resulting LSPR λ_max_, full width at half-maximum (fwhm), and absolute LSPR intensity of the tip-plasmon modes of (C) are plotted against the [Ag^+^]/[AA] molar ratio. Solid lines have been added to facilitate visualization of the general trend.

The extinction profile of plasmonic nanostars is governed by a complex interplay of structural and chemical factors, therefore necessitating a holistic interpretation of changes in LSPR peak shifts, broadening, and intensity variations. In a previous study,^12^ we devised a synthetic method based on the controlled overgrowth of gold onto preformed AuNSt. We observed that the blue shift of the tip-plasmon resonance band primarily results from a reduction in tip length and a decrease in the half angle, with the latter having a significantly more substantial impact on the LSPR displacement (Figure 1A).^9,12^

In this current study, the plasmonic response of the colloidal particles is also influenced by the change in elemental composition.^49,50^ As silver is deposited onto AuNSt, the increasing shell thickness alters the dielectric environment and enhances scattering, thereby simultaneously influencing the intensity and position of the LSPR peaks. Notably, unlike gold, silver does not experience significant resonance damping in the visible range with wavelengths below 600 nm, allowing for sharper and more intense plasmonic resonances in this spectral region.^51^

The silver coating of nanostars is a highly sensitive process governed by multiple parameters that collectively influence the uniformity, colloidal stability, and plasmonic properties of the resulting nanostructures. To tackle the complex challenge of optimizing the silver deposition, we employed UV–vis spectroscopy as a rapid, real-time, and nondestructive characterization tool to monitor how synthesis parameters affect the averaged optical properties of the colloidal suspension. Specifically, analyzing the position, width, and intensity of LSPR peaks provides indirect insights into morphological variations of the nanostars. The optimization process was carried out progressively to achieve the following goals:

1) Estimate the optimal Ag^+^ concentration and silver nitrate/ascorbic acid molar ratio ([Ag^+^]/[AA]) to achieve complete silver reduction on gold surfaces without external nucleation. This step also involved optimizing the ethanol-to-water volume ratio (EtOH/H_2_O). The goal was to obtain a tip-plasmon resonance with the highest intensity, the narrowest bandwidth, and the largest blue shift, supporting uniform silver coating within 24 h of synthesis.
2) Determine the optimum citrate anion concentration to guarantee colloidal stability.
3) Adjust the AuNSt concentration and refine the [Ag^+^]/[AA] ratio to enable full plasmon tunability below the 633 nm threshold
4) Finally, fine-tuning of the LSPR across the entire ∼ 600–800 nm range.

#### Preliminary Optimization of Ag^+^ and AA Concentrations

The first preliminary step of our iterative optimization process focused on estimating the minimum concentration of AA required to reduce the given amount of silver precursor onto a fixed number of AuNSt while avoiding silver nucleation in the bulk.

Initially, AuNSt dispersed in ethanol were combined with aqueous solutions of AA and AgNO_3_ to create an ethanol/water mixture with a volume ratio of 1:1. The final silver concentration was maintained at 4.75 × 10^–4^ M, with a fixed [Ag^+^]/[Au^0^] ratio of 5 (i.e., [Au^0^] = 9.5 × 10^–5^ M). The citrate concentration was initially set at 2.5 × 10^–4^ M, in agreement with the typical concentration used to stabilize small gold nanoparticles synthesized via sodium borohydride reduction of gold salt.^52,53^ At this concentration, citrate anions provided sufficient electrostatic stabilization to the spherical nanoparticles, while avoiding electrolyte-induced aggregation.

The AA content was systematically varied to adjust the [Ag^+^]/[AA] ratio from 75 to 7.5. After mixing, samples were left undisturbed 24 h before characterization. The extinction spectra (Figure 1B) exhibit a pronounced and continuous blueshift of the tip-plasmon resonance with increasing AA concentration (Figure S2), suggesting an incomplete reduction of silver ions even at elevated AA levels. Additionally, for [Ag^+^]/[AA] < 15, a new spectral contribution around ∼ 400 nm appeared, indicating silver nucleation in the bulk of the medium.

Based on these initial findings, we repeated the experiment with lower silver concentrations ([Ag^+^] = 5 × 10^–5^ M) and screened the [Ag^+^]/[AA] ratio in the 2.8–1.4 range (Figure S3A). Under these conditions, the LSPR λ_max_ reaches a plateau for [Ag^+^]/[AA] ratio below 1.8 (Figure S3B) while LSPR intensity peaked in the approximately 1.5–1.6 molar ratio range. Importantly, no new spectral contribution around 400 nm was observed, indicating that external silver nucleation was successfully prevented. These results indicate that most silver ions were effectively reduced onto the AuNSt surfaces. Thus, we selected [Ag^+^] = 5 × 10^–5^ M and a [Ag^+^]/[AA] ratio = 1.5 for further studies. Nonetheless, under these conditions, the tuning of the LSPR below ∼ 650–670 nm proved difficult.

#### Optimization of the Solvent Composition

The solubility of ascorbic acid decreases with decreasing solvent polarity, as it occurs in ethanol-rich environments.^54^ Consequently, its redox potential is also weakened as the ethanol content increases,^55^ which, in turn, may promote a more controlled silver growth by suppressing rapid bulk nucleation. On the other hand, a higher ethanol fraction can also weaken the electrostatic stabilization afforded by citrate anions, potentially leading to partial colloidal aggregation.

Figure 2A presents the extinction spectra for colloids synthesized at varying EtOH/H_2_O volume ratios from 0.12 to 183. The tip-plasmon resonances reach the maximum intensity and exhibit the narrowest bandwidth within the 2 to 4 volume ratio range (Figure 2B). For lower ethanol fractions, a more pronounced blue shift of the plasmon maxima is observed; however, this shift is accompanied by significant spectral broadening and reduced intensity. These effects suggest a notable rounding of the branched AuNSt morphology upon silver deposition. Conversely, at higher ethanol fractions, the tunability of the LSPR becomes increasingly restricted after 24 h. Under the lowest water content conditions, no appreciable shift in the tip-plasmon resonance is detected, while the emergence of a new spectral feature around ∼ 400 nm indicates silver nucleation in the bulk. Based on the results illustrated in Figure 2B, we therefore selected an EtOH/H_2_O volume ratio of 3 as the optimal condition for achieving controlled silver deposition.

**Figure 2:**
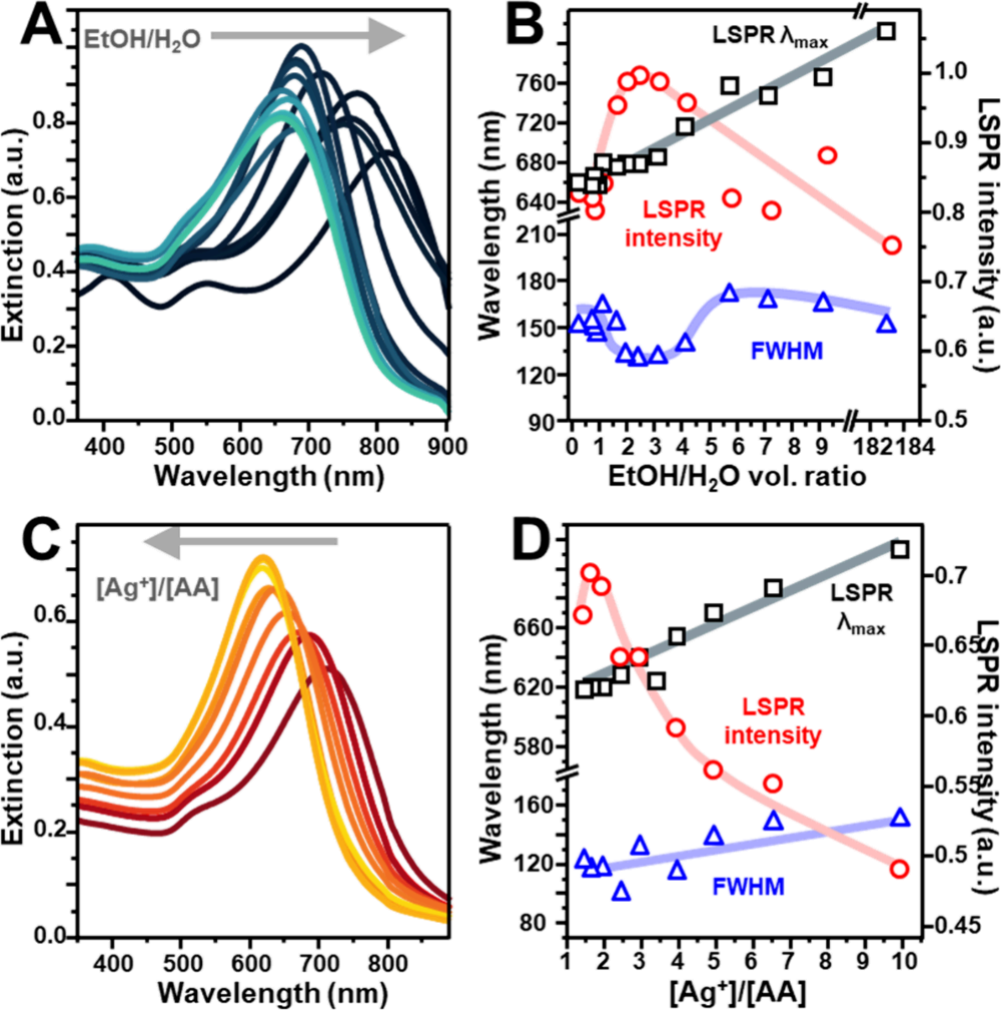
(A) Extinction spectra of AuNSt@Ag obtained under the following growth conditions: [Au^0^] = 9.5 × 10^–5^ M, [Ag^+^] = 5 × 10^–5^ M, [citrate] = 2.5 × 10^–4^ M, [AA] = 3.3 × 10^–5^ M. The EtOH/H_2_O volume ratio varied from 0.12 to 183. (B) Tip-plasmon spectral features as a function of the EtOH/H_2_O volume ratio. Solid lines have been added to facilitate visualization of the general trend. (C) Extinction spectra of AuNSt@Ag obtained under the following growth conditions: [Au^0^] = 6.6 × 10^–5^ M, [Ag^+^] = 5 × 10^–5^ M, [citrate] = 3 × 10^–4^ M, and EtOH/H_2_O volume ratio 3:1. AA concentration was varied to yield [Ag^+^]/[AA] ratios from 1.5 to 10. (D) Tip-plasmon spectral features as a function of the [Ag^+^]/[AA] ratio. Solid lines have been added to facilitate visualization of the general trend.

#### Role of Citrate Anions as Surface Stabilizing Agent

While keeping all other parameters constant, we subsequently investigated the effects of sodium citrate concentrations on the extinction spectra (Figure S4A). In the absence of citrate, the tip-plasmon resonance exhibits weak intensity and a very broad feature, suggesting that nanoparticle aggregation has occurred. As the citrate concentration increases, the plasmon band progressively narrows and gains intensity, reaching its maximum at ∼ 3 × 10^–4^ M sodium citrate (Figure S4B). Beyond this concentration, the LSPR shifts markedly toward the infrared region. Given that all samples contain the same silver precursor concentration, the reduced plasmon shift at higher citrate concentrations suggests that less silver is deposited on the nanostar tips. This implies that while citrate is crucial for colloidal stabilization, excessive amounts may hinder the deposition of silver ions onto the gold surface. Thus, we maintained the sodium citrate concentration of 3 × 10^–4^ M as the optimal value.

#### Reduction of AuNSt Concentration for Improved LSPR Tunability and Refinement of Ag^+^/AA Molar Ratio

Following the first optimization steps, we proceeded to refine the synthesis of AuNSt@Ag colloids under the updated EtOH/water volume ratio of 3 and with the goal of expanding tip-plasmon resonance tunability down to ∼ 600 nm. To this end, we reduced the number of AuNSt in the medium from [Au^0^] = 9.5 × 10^–5^ M to 6.6 × 10^–5^ M, while keeping the concentration of silver ions constant at 5 × 10^–5^ M ([Ag^+^]/[Au^0^] = 0.76). The [Ag^+^]/[AA] ratio was then varied from 10 to 1.7 by adjusting the AA concentration (Figure 2C).

A gradual blue shift of the plasmon maxima was observed as the [Ag^+^]/[AA] ratio was decreased below 10 (Figure 2D). In the [Ag^+^]/[AA] range of 2.0–1.7, LSPR λ_max_ approached a constant value of ∼ 620 nm while maximizing its intensity (Figure 2D). These results suggest that within this molar ratio range, the majority of silver ions were successfully reduced onto the gold surfaces.

To further investigate the stability and temporal evolution of the synthesized colloids, we also monitored the time-dependent changes in the extinction spectrum of colloids obtained with [Ag^+^]/[AA] = 1.7. After 2 days, the LSPR λ_max_ exhibited a slight shift to 617 nm, followed by a more pronounced shift to 606 nm after 2 weeks (Figure S5A). This latter shift was accompanied by noticeable broadening of the bandwidth, which may be attributable to morphological reshaping. These findings confirm that the vast majority of Ag^+^ ions were reduced within the first 24 h. Conversely, for [Ag^+^]/[AA] = 10, a more substantial LSPR shift of over 60 nm was observed over the same period (Figure S5B).

Thus, after this refinement step, we identified the optimized set of conditions: [Au^0^] = 6.6 × 10^–5^ M, [Ag^+^] = 5 × 10^–5^ M, [AA] = 3 × 10^–5^ M, EtOH/water volume ratio 3:1, and [Citrate] = 3 × 10^–4^ M. These concentrations correspond to [Ag^+^]/[AA] and [Ag^+^]/[Au^0^] molar ratios of 1.7 and 0.76, respectively.

#### Role of Residual PVP

Polyvinylpyrrolidone (PVP) was used as both a reducing and stabilizing agent during AuNSt seed synthesis. To minimize interference from residual PVP during subsequent silver overgrowth, the number of washing cycles was limited to seven in order to ensure full colloidal stability. To evaluate the impact of residual PVP on silver deposition, we conducted two sets of experiments in which silver coating was performed on AuNSts: (i) subjected to different numbers of centrifugation cycles (Figure S6A), and (ii) in the presence of known concentrations of PVP added to the reaction medium (Figure S6B). These results confirm that higher concentrations of polymer molecules are likely to hamper silver deposition onto the gold surface. Ultimately, AuNSt washed seven times were identified as the optimal compromise, minimizing aggregation risk while ensuring sufficient removal of PVP for subsequent silver overgrowth. For a more detailed discussion on this topic, see Supporting Information, page S7.

#### Fine-Tuning of LSPR across the Entire Spectral Range of Interest

Successful tuning of the tip-plasmon resonance was ultimately achieved by simply varying the concentrations of Ag^+^ and AA while maintaining the corresponding [Ag^+^]/[AA] ratio = 1.7. Figure 3A presents the normalized extinction spectra of AuNSt@Ag colloids synthesized with [Ag^+^] ranging from 0.5 × 10^–5^ M to 7.5 × 10^–5^ M. The increase of the silver precursor concentration led to the progressive blue shift of the plasmon band maxima across the full 600–800 nm range without evidence of free silver nucleation throughout the entire concentration series. This was accompanied by the increment of LSPR intensity and the narrowing of its bandwidth (Figure 3B). Similar general trends were also observed in numerical simulations of the extinction cross sections for AuNSt@Ag structures with increasing silver shell thickness (Figure S7). Overall, these results suggest a corresponding increase in silver deposition on the AuNSt surface while largely preserving the characteristic star-shaped morphology, a hypothesis which is corroborated by TEM analysis (Figure S8 and S9). An exception was observed at the highest Ag^+^ concentration, where the resulting AuNSt@Ag colloids (λ_max_ = 600 nm) exhibit a decrease in LSPR intensity, peak broadening, and the emergence of an extended shoulder at longer wavelengths. Based on the TEM images illustrated in Figures 4, S8, and S9, we likely attribute this deviation to a significant modification of the external morphology caused by extensive Ag deposition, which reduces the sharpness of the silver-coated tips.

**Figure 3:**
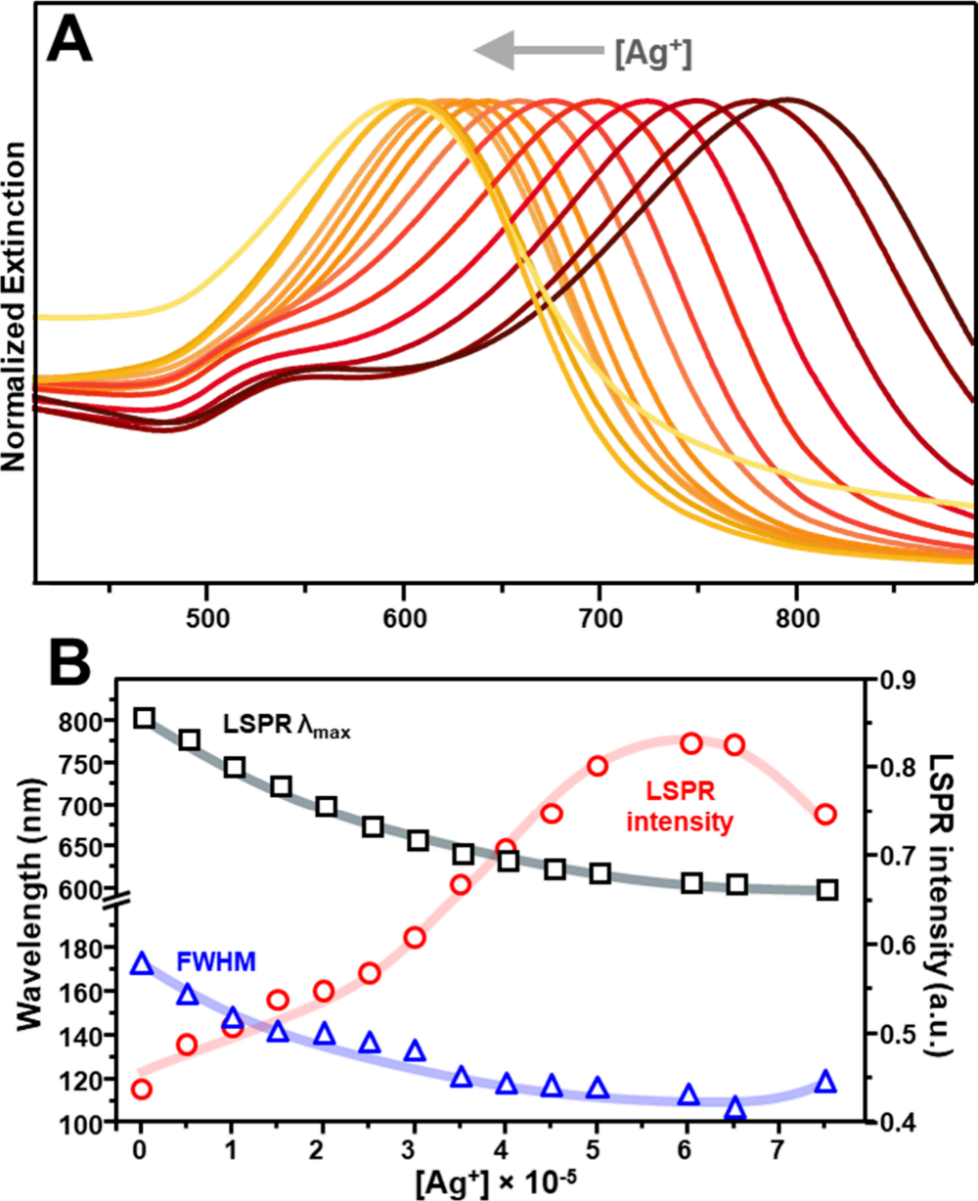
(A) Normalized extinction spectra of AuNSt@Ag obtained under the following growth conditions: seven times cleaned AuNSt, [Au^0^] = 6.6 × 10^–5^ M, [citrate] = 3 × 10^–4^ M, and EtOH/H_2_O volume ratio 3:1. The [Ag^+^] concentration was varied from 5.0 × 10^–6^ to 5.0 × 10^–5^ M, while the [AA] concentration was adjusted accordingly to maintain a constant [Ag^+^]/[AA] ratio of 1.7. (B) Tip-plasmon spectral features as a function of Ag^+^ concentration. Solid lines have been added to facilitate visualization of the general trend.

**Figure 4:**
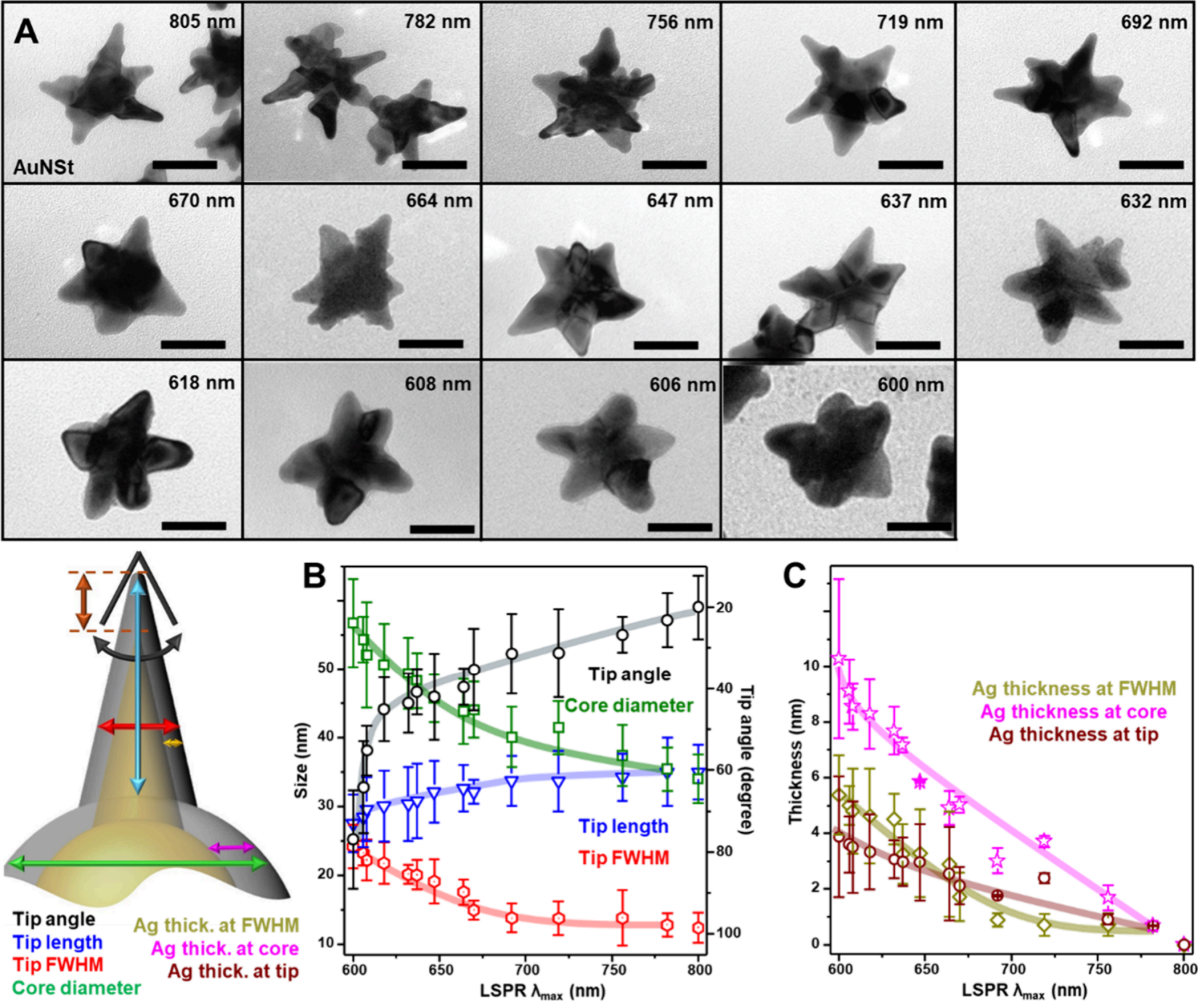
(A) Representative TEM images of AuNSt and AuNSt@Ag, corresponding to the colloidal samples from Figure 3 (scale bars: 50 nm). (B) Morphological parameters of the nanoparticles (tip angle, tip length, tip full width at half-maximum (fwhm), and core diameter) as a function of their LSPR λ_max_. (C) Silver thickness at different structural regions of the nanostars (core, tips, and fwhm) as a function of their LSPR λ_max_. Solid lines have been added to facilitate visualization of the general trend. The number of morphologically analyzed particles is approximately 50 for each sample.

### Morphological and Compositional Characterization

The UV–vis spectroscopy screening facilitated the refinement of the synthetic methodology by providing an indirect correlation between the plasmonic properties and the average morphology of AuNSt@Ag. In this second part of the study, advanced characterization techniques are employed to detail specific morphological and compositional changes of AuNSt@Ag colloids with different degrees of silver coating.

Figure 4A and Figure S8–S9 illustrate representative transmission electron microscopy (TEM) images of the colloidal samples corresponding to the extinction spectra shown in Figure 3 **A.** Across all samples, the nanoparticles exhibit well-defined tips, even when extensively coated with silver, demonstrating a highly tunable plasmonic response with minimal morphological rounding. This preservation of geometrical integrity aligns with the LSPR characteristics observed in the extinction spectra.

The morphological evolution of nanostar geometry was quantified at a single particle level in terms of (1) tip length, (2) tip full width at half-maximum (fwhm), (3) tip angle, (4) core diameter, and (5) tip-to-tip length (Figure 4B and Figure S10). A linear increase is observed in tip width and core diameter as silver deposition progresses, suggesting that silver growth occurs across all gold surfaces from the very beginning of the process. Simultaneously, the tip length is reduced only by ∼ 15% when comparing uncoated gold nanostars to AuNSt@Ag (λ_max_ = 606 nm), despite the approximately 35% increase in core diameter. This is also accompanied by a × 1.8 times increase of the tip width, a morphological change which is coherently mirrored by a rapid increase in tip angle.

Due to the distinct electron scattering properties of gold and silver, bright-field TEM imaging reveals differential mass–thickness contrast, enabling estimation of silver shell thickness across various structural regions. These values, shown in Figure 4C, confirm the trends observed from overall particle dimensions, indicating a steady increase in silver layer thickness with increasing deposition, where the growth occurs more rapidly at the core than at the tips.

These findings are consistent with the known preference of silver to accumulate at the core, where surface energy is lower, and growth is diffusion-controlled.^56^ Nonetheless, our optimized synthesis method efficiently mitigates this intrinsic tendency, promoting a more uniform silver deposition. This results in a minimal loss of tip sharpness and preservation of star-like morphology, even after extended silver deposition.

Scanning transmission electron microscopy (STEM) analysis in combination with spectroscopic mapping by energy-dispersive X-ray (EDX) spectroscopy was carried out on two representative AuNSt@Ag samples with λ_max_ centered at 780 and 620 nm (Figure 5 and Figure S11). Elemental mapping results confirm the formation of a core–shell structure, where a homogeneous Ag shell uniformly coats the Au nanostar core. Notably, the successful coating of the gold tips is observed not only in nanostars with higher Ag content but also in those with the lowest Ag content. This finding is particularly significant, as it demonstrates that the coating process occurs across the entire gold surface, including the tips, even when the amount of Ag is very low. Furthermore, EDX analysis reveals an elemental Au/Ag composition ratio of 89:11 and 54:46 for AuNSt@Ag samples with λ_max_ = 780 nm and λ_max_ = 620 nm, respectively.

**Figure 5:**
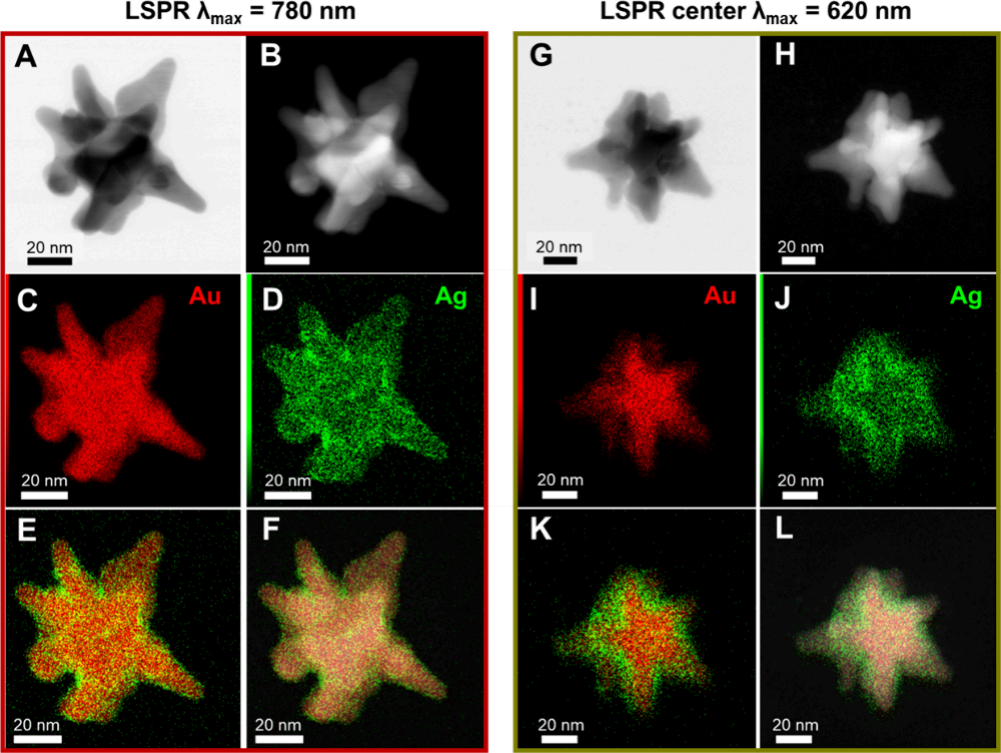
(A, G) Bright-field scanning transmission electron microscopy (BF-STEM) and (B, H) high-angle annular dark-field scanning transmission electron microscopy (HAADF-STEM) images of AuNSt@Ag (λ_max_ = 780 and 620 nm), highlighting the Z-contrast between gold (Au) and silver (Ag). (C, D, I, J) Elemental maps obtained via energy-dispersive X-ray (EDX) analysis show individual distributions of Au (C, I) and Ag (D, J). (E, F, K, L) Overlay of Au and Ag elemental maps without (E, K) and with (F, L) the HAADF-STEM image, confirming that Ag forms a shell around Au while maintaining the nanostar morphology.

High-Resolution Transmission Electron Microscopy (HRTEM) images and corresponding Fast Fourier Transform (FFT) patterns (Figure 6) were obtained at different points along the tip of two individual AuNSt@Ag nanoparticles (from colloids with λ_max_ = 780 and 620 nm, respectively). The selected regions include the end (red square), the middle (purple square), and the side of the tip (green square). In both cases, the continuous fringe patterns confirm the single-crystalline nature of the tips, with a predominant [011] growth direction, as indicated by the white arrows. The absence of twin planes in the individual tips further supports this hypothesis. Such a crystallographic orientation implies exposure of {110} facets at the tip, which are known to have higher surface energies compared to {111} and {100} facets. This is consistent with the expected behavior of face-centered cubic (fcc) lattices.^45,57^

**Figure 6:**
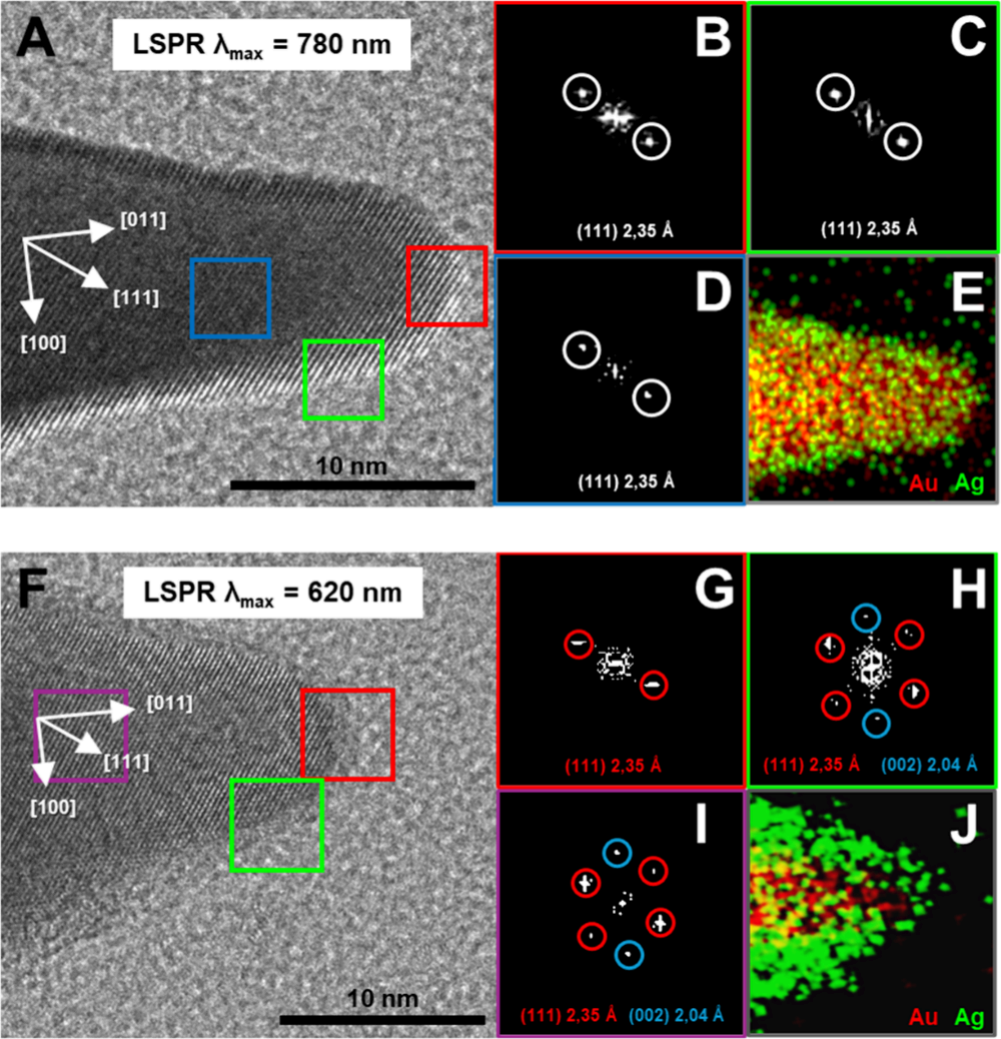
(A, F) High-resolution transmission electron microscopy (HRTEM) images of AuNSt@Ag nanoparticles (from colloids with λ_max_ = 780 and 620 nm, respectively), highlighting the [011] growth direction at different points along the nanostar tips. (B–D, G–I) Corresponding fast Fourier transform (FFT) patterns from the marked square regions in (A, F), revealing the characteristic lattice planes of a face-centered cubic (FCC) crystalline structure. (E, J) Energy-dispersive X-ray (EDX) analysis of the tip, confirming the distribution of Au and Ag.

FFT analysis of the tip end and side regions reveals diffraction patterns corresponding to (111) planes (red square), and (111) plus (002) planes (green square), in agreement with crystalline silver. This is further supported by EDX elemental mapping and the brighter contrast in the HRTEM images. In contrast, the midtip region (purple square) exhibits the same crystallographic planes but corresponds to a buried gold core coated by a silver layer. Together, these observations indicate that, in the absence of stacking faults or twin planes, silver is deposited epitaxially onto the gold tips, with preferential growth on the lower-energy (111) and (100) facets.^34^

It is well-known that high-curvature regions such as tips and edges exhibit higher surface energies than flat facets, making Ag nucleation at these sites less favorable under typical conditions. However, we suggest that the slow and controlled reduction kinetics employed in our method ensure a steady and limited supply of Ag^0^ atoms. This allows sufficient time for surface diffusion, promoting uniform redistribution of Ag atoms across the AuNSt surface. As a result, Ag deposition occurs conformally, even at high-energy tip regions, without inducing uncontrolled nucleation or morphological disruption.^58,59^

### SERS Characterization

Building on the optical and structural optimization described above, we next evaluated the surface-enhanced Raman scattering (SERS) performance of the AuNSt@Ag colloids. A simple thiolated molecule such as thiophenol (TP) was selected as a model probe molecule. TP is widely employed as a nonresonant probe molecule for assessing the SERS properties of plasmonic nanomaterials. Owing to its strong affinity for gold and silver surfaces via the thiol functional group, TP firmly attaches covalently to both metallic surfaces (Au and Ag), yielding stable and intense spectra with sharp and well-defined features.

Furthermore, the absence of additional functional groups prevents unwanted interactions with chemical species in the medium, which could otherwise alter the spectral response or introduce variability in adsorption behavior. This is crucial to enabling a more direct comparison of the intrinsic plasmonic efficiency of the nanostructures, independent of differences in surface affinity or molecular orientation.

To ensure meaningful cross-comparisons of SERS responses, the synthesized samples were not subjected to postcentrifugation or redispersion steps, which would lead to unpredictable variations in colloidal concentration. Instead, an aliquot of ethanolic TP solution was directly added to the nanoparticle reaction medium, where all diluted colloidal samples contained an equivalent number of AuNSt seeds.

Figure 7A presents the SERS spectrum of TP-functionalized AuNSt@Ag (λ_max_ = 640 nm). SERS measurements were conducted using a macrosampling setup, yielding spatially averaged signals from a large number of nanoparticles in suspension. In addition to Raman bands of the ethanol solvent (e.g., 885, 1052, 1094, and 1454 cm^–1^), characteristic TP features appear at 419, 1000, 1024, 1076, and 1577 cm^–1^.^60^ The TP band at 1577 cm^–1^, attributed to aromatic ν­(CC) modes,^60^ does not overlap with any ethanol Raman signals, making it an ideal marker for quantitative analysis. Given that ethanol concentration remains constant across all samples, the solvent band at 1454 cm^–1^ was used as an internal standard to mitigate fluctuations arising from experimental variability, thereby improving the reliability of the data. Consequently, the intensity ratio I_1577_/I_1454_ (i.e., ratiometric SERS intensity) was employed to monitor changes in SERS signal across different bimetallic colloids.

**Figure 7:**
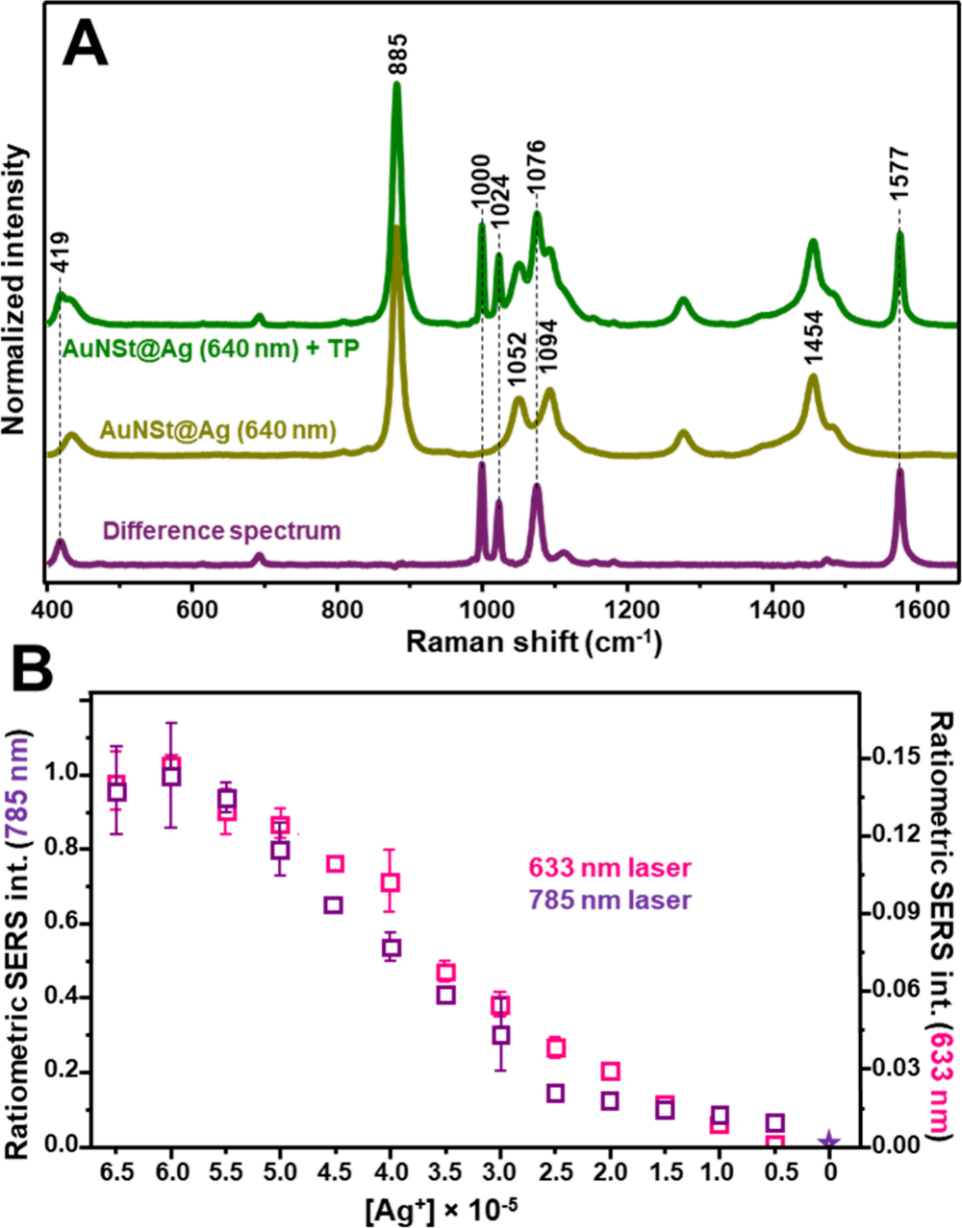
(A) SERS spectra of AuNSt@Ag nanoparticles (λ_max_ = 640 nm) before and after the addition of TP at a final concentration of 3 × 10^–7^ M (excitation wavelength = 785 nm). The difference spectrum is included to clearly reveal the characteristic vibrational fingerprint of TP. (B) Ratiometric SERS intensity (*I*_1570_/*I*_1454_) plotted as a function of silver concentration for both 633 and 785 excitation lines. AuNSt@Ag nanoparticles were functionalized with a mixed MUA/TP layer. The results represent the averaged ratiometric SERS intensities with corresponding standard deviations calculated from triplicate measurements (*N* = 3) performed on independently prepared aliquots.

An additional critical factor in the relative quantitative SERS analysis is the prevention of nanoparticle clustering, which may result from TP adsorption onto metallic surfaces, that would significantly alter the local electromagnetic fields at the nanoparticle tips.^9^ A screening study was conducted to address this issue in which AuNSt@Ag particles were exposed to TP concentrations ranging from 5 × 10^–8^ M to 5 × 10^–6^ M. The samples were left undisturbed for 2 h before optical characterization to ensure comparable, if not complete, thiol surface coverage. Extinction spectra revealed meaningful profile changes for probe concentrations ≥ 3 × 10^–7^ M, indicating that nanoparticle aggregation had already occurred to varying degrees (Figure S12). Conversely, the SERS signals recorded at concentrations below this threshold were very weak, making them poorly suited for reliable monitoring of the entire set of colloidal samples.

Therefore, to acquire sufficiently intense TP signals from nonaggregated particles, we adopted a previously reported methodology for the fabrication of colloidally stable SERS-encoded nanoparticles.^47^ Bimetallic nanostars were prefunctionalized with a submonolayer of mercaptoundecanoic acid (MUA), to ensure high colloidal stability during subsequent TP adsorption (Figure S13). Indeed, the covalent binding of MUA simultaneously stabilizes the nanoparticles through steric repulsion, provided by its long aliphatic chain, and electrostatic repulsion from its terminal carboxylic group. Importantly, due to its aliphatic nature, MUA exhibits an almost negligible Raman cross-section compared to aromatic compounds, thereby avoiding vibrational interference in the SERS spectra. It is worth noting that, even assuming full adsorption, the total amount of MUA + TP remains well within the submonolayer regime (see page S15 of Supporting Information for a detailed explanation). This ensures a reliable, direct comparison of the SERS response across nanostars with slightly different surface areas due to varying Ag coating thicknesses. In this way, any observed differences in SERS intensity can be confidently attributed to the optical efficiency of the nanoparticles rather than to variations in the number of TP molecules adsorbed per particle.

In Figure 7B, ratiometric SERS intensities I_1577_/I_1454_ of bimetallic nanostars modified with a mixed MUA/TP layer are plotted against the silver concentration. For 633 and 785 nm excitation wavelengths, silver-coated gold nanostars exhibit a clear trend: the SERS efficiency progressively rises as the silver content is increased, reaching a maximum around 6.0 × 10^–5^ M. This corresponds to colloids with LSPR λ_max_ falling within the ∼ 605–615 nm range (Figure S14). Notably, for the 785 nm laser line, the most efficient AuNSt@Ag material (λ_max_ = 608 nm) drastically outperforms the original AuNSt colloids (λ_max_ = 815 nm) by factors of approximately 140×, However, the tip-plasmon resonance of the AuNSt seeds is significantly red-shifted relative to the 785 nm excitation line. According to both theoretical models and experimental studies on SERS,^12,51^ maximum electromagnetic enhancement typically occurs when the LSPR is slightly blue-shifted relative to the excitation laser. To test this optimal resonance condition and allow for a meaningful comparison between monometallic and bimetallic systems, we synthesized AuNSt colloids with a tip-plasmon resonance centered at ∼ 780 nm. Under these conditions, we observed a clear increase in the TP signal as compared to the AuNSt seeds (approximately 1.75 times). Nonetheless, the corresponding ratiometric value remained approximately 80 times lower than the maximum signal recorded for the best-performing AuNSt@Ag colloids. Such improvements significantly exceed previous reports for silver-coated gold nanostars, which typically demonstrated enhancement in the range of 5 to 10.^18,30,31,36,37,40,41^

Our results indicate that the same class of AuNSt@Ag colloids simultaneously exhibit maximum SERS efficiency under both lasers, even with a substantial mismatch between the LSPR and the excitation wavelength (especially under 785 nm excitation, see Figure S14). This behavior contrasts markedly with that observed in our previous study,^12^ where the SERS response of monometallic AuNSt showed a strong correlation between the tip-plasmon maximum and the excitation wavelength. In that earlier study, maximum SERS enhancement was observed when the LSPR was slightly red-shifted relative to the excitation line.^12,61^ In the case of our AuNSt@Ag nanostructures, the SERS results indicate that a fine spectral matching between the excitation wavelength and the LSPR peak is not the primary factor governing SERS performance. Instead, it is the presence of a silver shell, with its superior plasmonic properties, which appears to play the dominant role. Indeed, analogous findings were also reported for conventional AuNSt@Ag colloids in which silver deposition is primarily concentrated at the central core of the gold nanostars, leaving the gold tips largely uncoated.^36^ In these structures, tip-enhancement effects were also found to increase with the progressive thickening of the silver shell, reaching a maximum before declining, regardless of the detuning of LSPR maximum from the excitation wavelength.^36^ Overall, our results reinforce the previous notion that the dominant factor influencing SERS performance in such bimetallic systems is not fine spectral alignment with the laser frequency, but rather the material composition and local morphology.

## Conclusions

In this study, we developed a robust and finely tunable synthesis strategy for silver-coated gold nanostars (AuNSt@Ag), enabling precise control over their plasmonic response while preserving their complex branched morphology, even at high silver loadings. Through systematic optimization of key experimental parameters, we achieved continuous modulation of the tip-plasmon resonance across the entire 600–800 nm spectral window, without inducing free silver nucleation.

High-resolution electron microscopy and elemental mapping confirmed that the silver shell forms uniformly across the nanostar surfaces—including the tips—even at minimal Ag deposition. This homogeneity in shell growth is crucial for preserving the sharp nanoscale features responsible for strong near-field electromagnetic enhancement.

A central and unexpected finding of our work is that a single nanostructure class (AuNSt@Ag with LSPR maxima around 605–615 nm) can simultaneously maximize SERS performance under two widely separated excitation wavelengths (633 and 785 nm). This result highlights the dominant role of localized field enhancement at silver-coated tips, which remains highly effective regardless of resonance alignment with the excitation source. Notably, our optimized bimetallic nanostructures exhibit up to a 140-fold enhancement compared to their gold nanostar seeds, and an 80-fold increase relative to optimized AuNSt with a tip-plasmon resonance aligned to the 785 nm laser, substantially outperforming previously reported silver-coated gold nanostars.

In conclusion, our synthetic strategy offers a scalable and reproducible platform for the design of high-efficiency plasmonic materials. These findings deepen the understanding of structure–function relationships in anisotropic bimetallic nanostructures and provide new opportunities for their application in ultrasensitive molecular sensing and photonic technologies.

## Supplementary Material



## Notes

Extinction spectra showing: the time evolution of AuNSt@Ag nanoparticles, their cleaning process, the influence of sodium citrate during silver overgrowth, and the spectra of AuNSt@Ag colloids functionalized with varying concentrations of TP and with MUA/TP mixtures; data on the variations in the LSPR λ_max_, fwhm, and absolute LSPR intensity of tip-plasmon modes for different silver coating thicknesses; numerical simulations and detailed corresponding data; additional TEM images of AuNSt@Ag nanoparticles, including electron tomography reconstructions; ratiometric SERS intensities plotted as a function of AuNSt@Ag LSPR for both excitation wavelengths (PDF)
