# Supplementary material for: Synthesis and Optimization of Highly Bright Silver-Coated Au Nanostars with Tunable Plasmonic Properties
Source: ACS Nanosci Au. 2025 Sep 11;5(6):469–81. doi: 10.1021/acsnanoscienceau.5c00075 (PMC12715626; doi:10.1021/acsnanoscienceau.5c00075)
Supplement: Supplementary file 1 [file ng5c00075_si_001.pdf]

Synthesis and Optimization of Highly Bright Silver-Coated Au Nanostars with Tunable Plasmonic Properties

Judith Peñas-Farré,[1] Xiaofei Xiao,[2] Vincenzo Giannini,[2,3,4] Xavier Mateos,[1] Luca Guerrini,[1,] and Nicolas Pazos-Perez[1,*]*

[1] Department of Physical and Inorganic Chemistry. Universitat Rovira i Virgili, Carrer de Marcel·lí Domingo 1, 43007 Tarragona, Spain.

[2] Technology Innovation Institute, Building B04C, Masdar City, Abu Dhabi P.O. Box 9639, United Arab Emirates

[3] Instituto de Estructura de la Materia (IEM-CSIC), Consejo Superior de Investigaciones Científicas, Serrano 121, 28006 Madrid, Spain

[4] Centre of Excellence ENSEMBLE3 sp. z o.o., Wolczynska 133, 01-919 Warsaw, Poland.

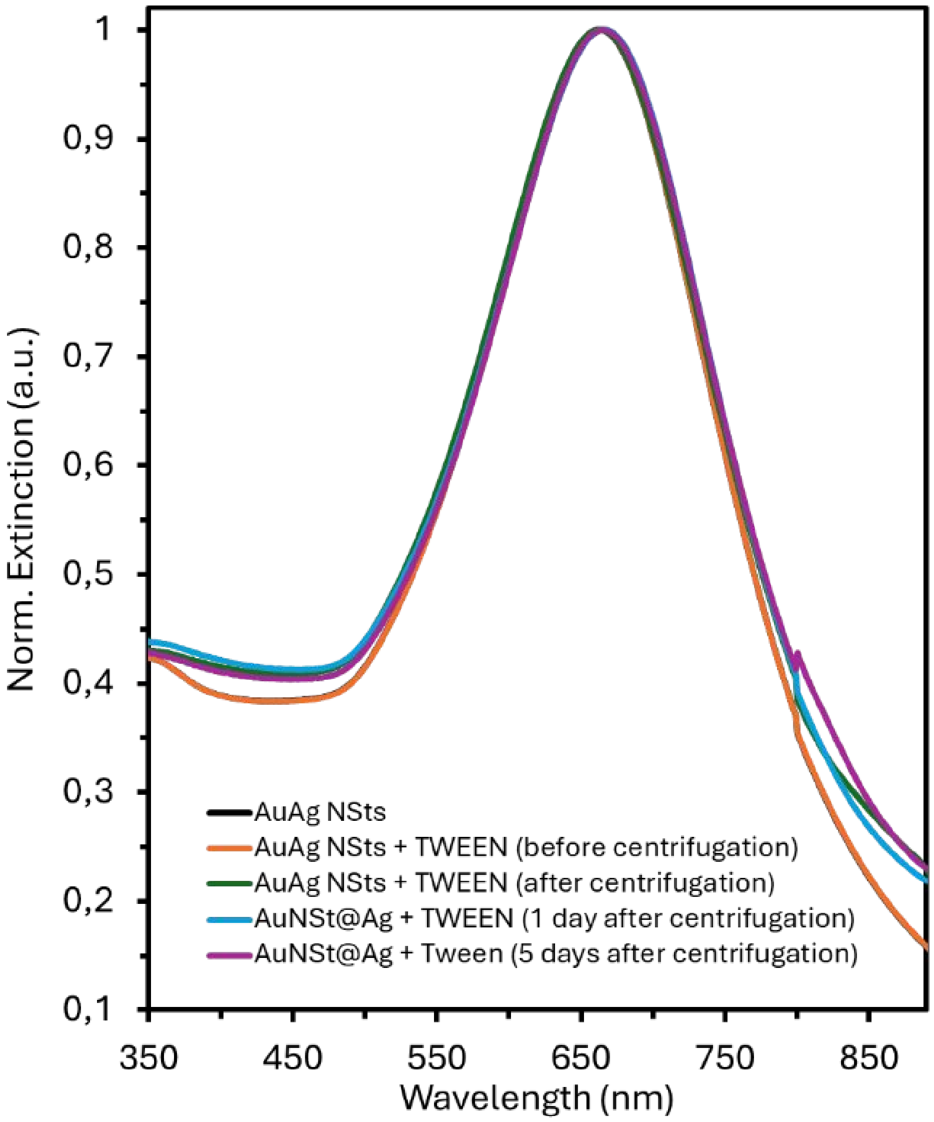

Figure S1. Extinction spectra of the as-prepared AuNSt@Ag colloids and the intermediate colloids obtained during the Tween-assisted purification process.

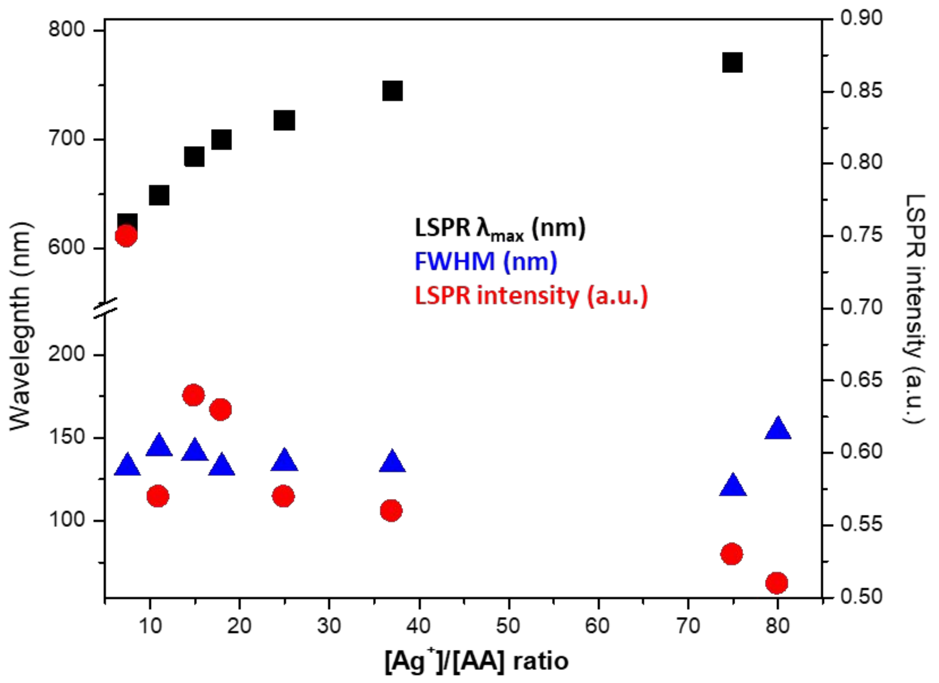

Figure S2. LSPR λ_{max}, full width at half-maximum (FWHM), and absolute LSPR intensity of the tip-plasmon modes of Figure 1B vs $[Ag^+]/[AA]$ ratio.

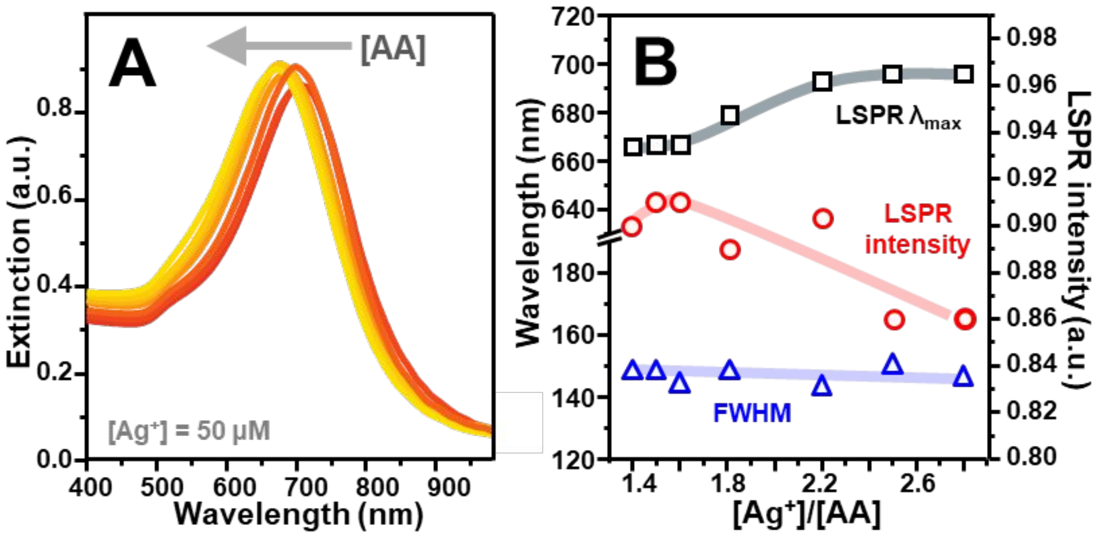

Figure S3. (A) Extinction spectra of AuNSt@Ag obtained under the following growth conditions: $[Au^0] = 9.5 \times 10^{-5}$ M, $[Ag^+] = 5 \times 10^{-5}$ M, [citrate] = 2.5×10^{-4} M, with an EtOH/H_2O volume ratio 1:1. The $[Ag^+]$/[AA] ratio was varied from 1.4 to 2.8. (B) The resulting LSPR λ_{max}, full width at half-maximum (FWHM), and absolute LSPR intensity of the tip-plasmon modes of (C) are plotted against the $[Ag^+]$/[AA] molar ratio. Solid lines have been added to facilitate visualization of the general trend.

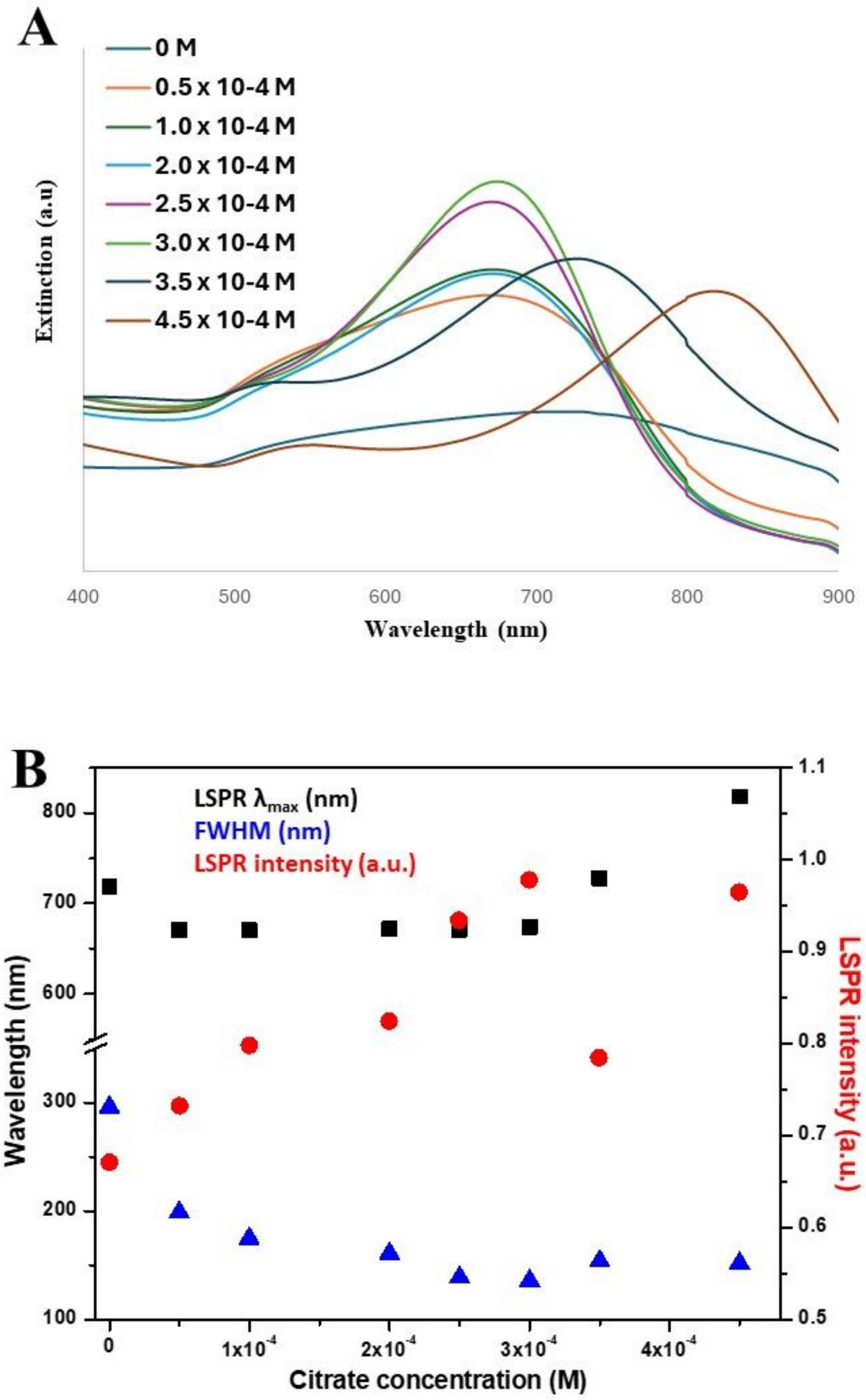

Figure S4. (A) Extinction spectra of AuNSt@Ag obtained under the following growth conditions: $[Au^0] = 9.5 \times 10^{-5}$ M, $[Ag^+] = 5 \times 10^{-5}$ M, $EtOH/H_2O$ volume ratio 3:1, and

[AA] = 3.3 × 10^{-5} M. The citrate concentration was varied from = 0 to 4.5 × 10^{-4} M. (B) Tip-plasmon spectral features as a function of the citrate concentration.

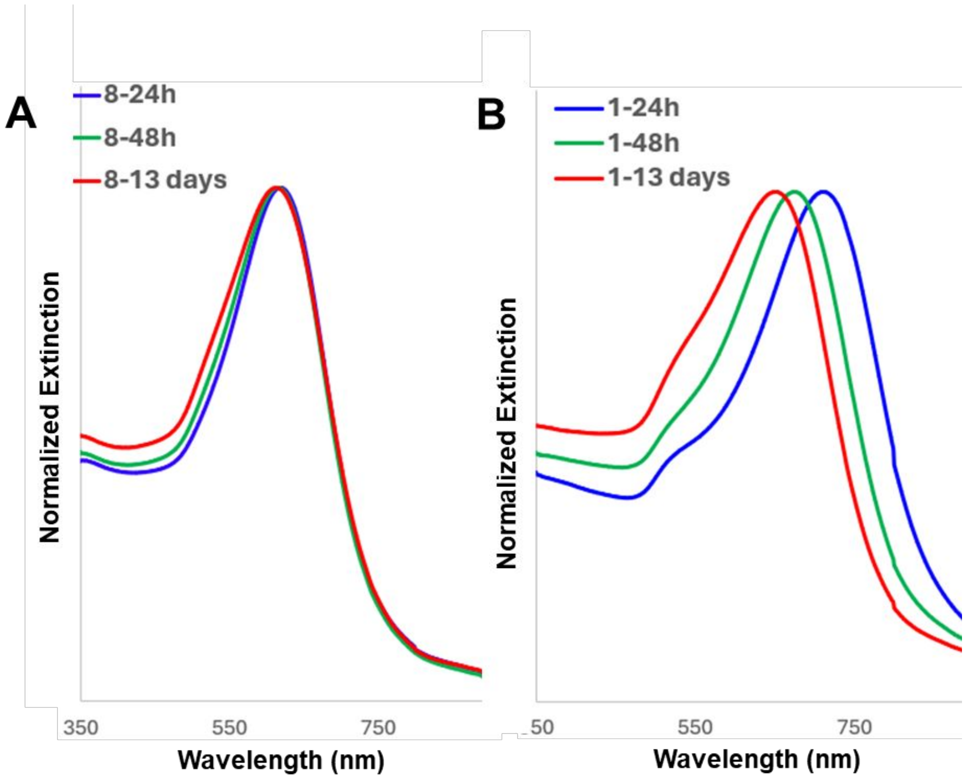

Figure S5. Time dependent evolution of extinction spectra of AuNSt@Ag obtained under the following growth conditions: [Au^0] = 6.6 × 10^{-5} M, [Ag^+] = 5 × 10^{-5} M, [citrate] = 3 × 10^{-4} M, and EtOH/H_2O volume ratio = 3. (A) [Ag^+]/[AA] = 1.7 and (B) [Ag^+]/[AA] = 1.7.

THE ROLE OF RESIDUAL PVP

Polyvinylpyrrolidone (PVP) acts as both a reducing and stabilizing agent in the synthesis of AuNSt seeds. To minimize interference from PVP molecules adsorbed on the gold surfaces during subsequent silver overgrowth, the synthesized AuNSt were subjected to multiple centrifugation cycles. However, when the number of centrifugation cycles exceeded nine, significant aggregation of the colloids was observed. Consequently, colloids washed seven times were selected for silver coating in order to balance effective PVP removal with colloidal stability.

Nonetheless, despite this extensive cleaning, residual PVP molecules are expected to remain adsorbed on the gold surfaces, as indirectly indicated by the preserved colloidal stability of the nanoparticles. Thus, to better evaluate the impact of residual PVP on silver deposition, two sets of experiments were conducted. First, silver overgrowth was performed on AuNSts subjected to varying numbers of centrifugation cycles (**Figure 2E**). The results showed that increasing the number of washing cycles from five to seven or eight led to a gradual blue shift of the localized surface plasmon resonance (LSPR) to approximately 620 nm, accompanied by an increase in intensity.

Second, silver coating was performed on seven-times-washed AuNSt in the presence of increasing concentrations of PVP added to the reaction medium (**Figure 2F**). As the PVP concentration increased from 0 to 3.5×10^{-4} M, the LSPR exhibited again a progressive red shift along with a decrease in intensity. These results confirm that a higher concentration of polymer molecules in the medium likely inhibits silver deposition onto the gold surfaces. Ultimately, AuNSt washed seven times were correctly identified as the optimal compromise as they minimize aggregation risk while ensuring sufficient removal of PVP for controlled silver overgrowth.

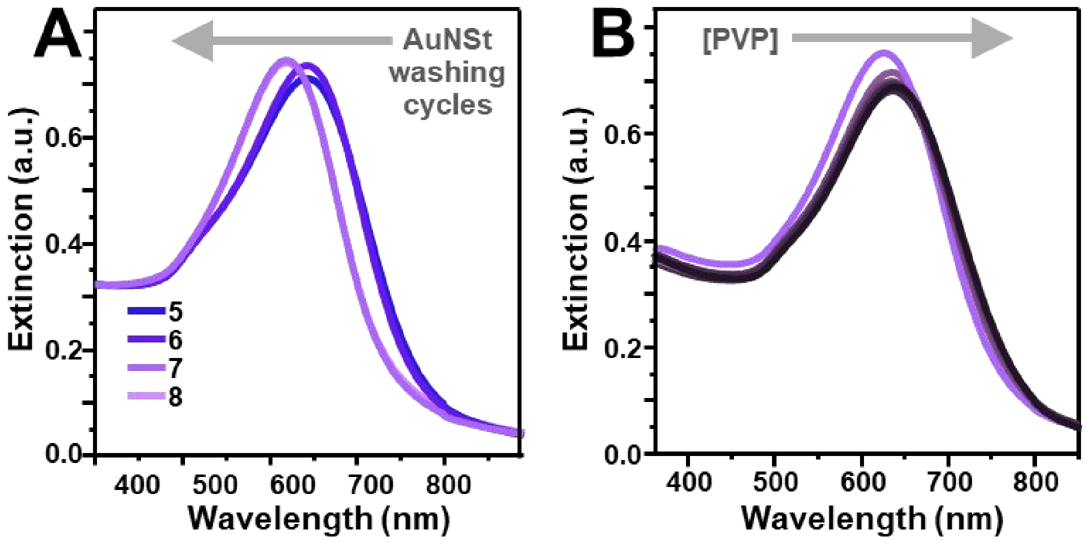

Figure S6. (A) Extinction spectra of AuNSt@Ag obtained from AuNSt seeds subjected to varying numbers of centrifugation cycles prior to silver coating. The growth conditions for AuNSt@Ag were the following: [Au^0] = 6.6×10^{-5} M, [Ag+] = 3×10^{-5} M, [citrate] = 3×10^{-4} M, and EtOH/H2O volume ratio 3:1. (B) Extinction spectra of AuNSt@Ag obtained from AuNSt seeds subjected to seven centrifugation cycles before silver coating. Additional PVP was introduced into the reaction medium, yielding concentrations ranging from 0 to 3.5×10^{-4} M.

NUMERICAL SIMULATIONS

To further investigate the observed plasmon shift, theoretical modeling was performed using the Total-Field Scattered-Field (TFSF) source and associated analysis tools within the Lumerical software suite to calculate the extinction cross section. The modeled nanostar structure comprises a central spherical gold core with six protruding tips of varying lengths and orientations, embedded in a medium with a refractive index of n = 1.36, is described in Figure S5A. The simulation parameters are summarized in corresponding Table. While the geometry of the gold nanostar core was held constant, the thickness of the silver shell was systematically varied. To better align with experimental observations and reduce computational complexity, certain geometric parameters were adjusted from the experimentally derived morphology. As shown in Figure S5B, the theoretical results of the corresponding extinction cross sections exhibit the same trend observed experimentally: a plasmonic blue-shift with increasing silver shell thickness. The broadening of the extinction spectra and the resonance shifts are primarily attributed to deviations between the idealized simulated geometry and the actual nanostar morphology, as well as to inherent size and shape heterogeneities among particles in the experimental samples.

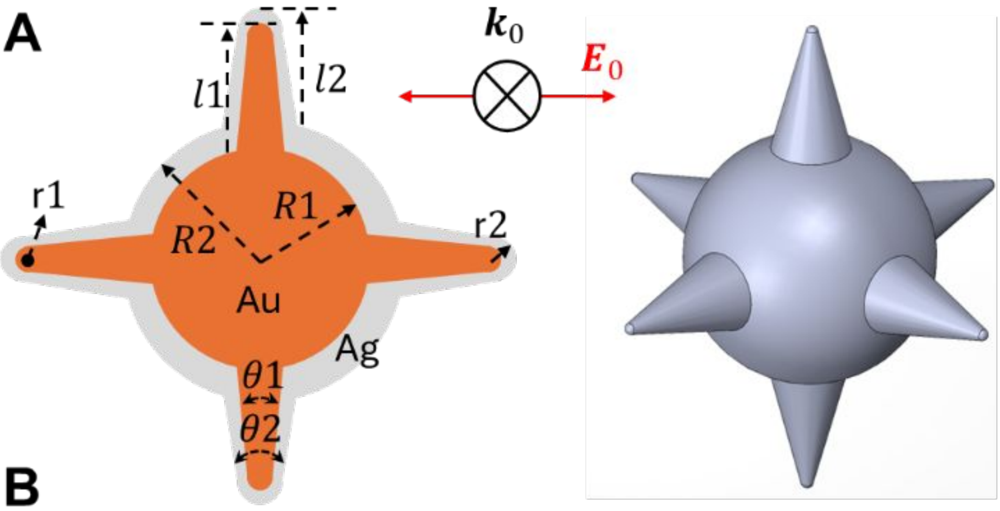

B

Case	$\theta1$(degs)	r1	R1	l1	$\theta2$ (degs)	r2	R2	l2	n_b
C1	20	2	17.5	36	50	3	35.5	20	1.36
C2	20	2	17.5	36	45	3	26	30	1.36
C3	20	2	17.5	36	40	3	23	32	1.36
C4	20	2	17.5	36	32	3	21	34	1.36
C5	20	2	17.5	36	27	3	19.5	35	1.36

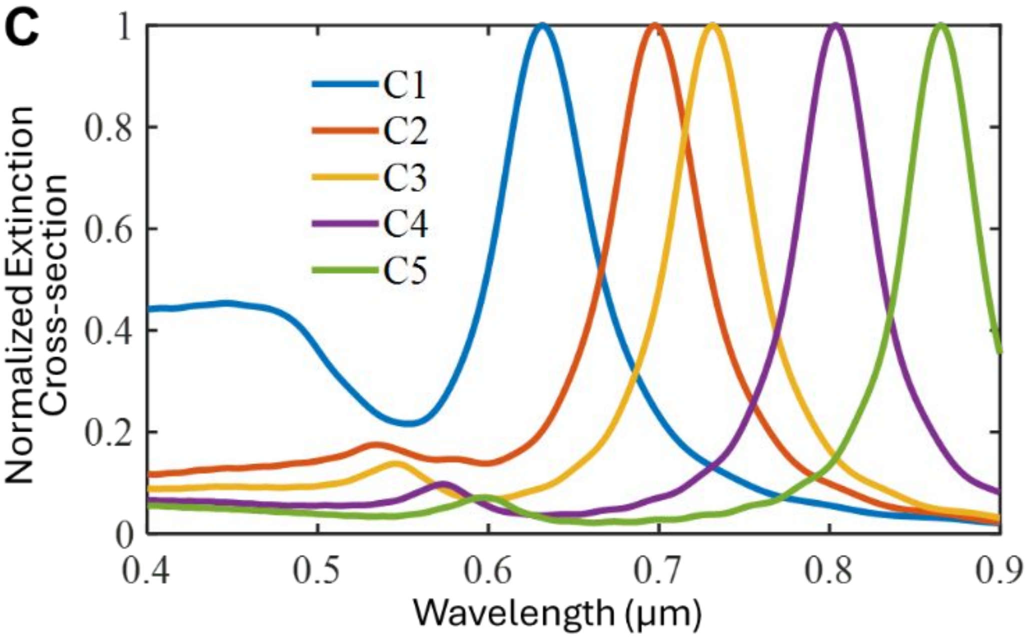

Figure S7. (A) Schematic of the nanostar geometry, which consists of a central spherical core with six protruding tips of varying lengths and orientations, all embedded in a medium with a refractive index of n = 1.36. Each nanostar was modeled as a hybrid structure composed of a gold nanostar core coated with a silver nanoshell. In all simulations, an x-polarized plane wave was incident normally along the negative z-axis. (B) Table summarizing the simulation parameters. (C) Normalized extinction cross sections of the modelled systems.

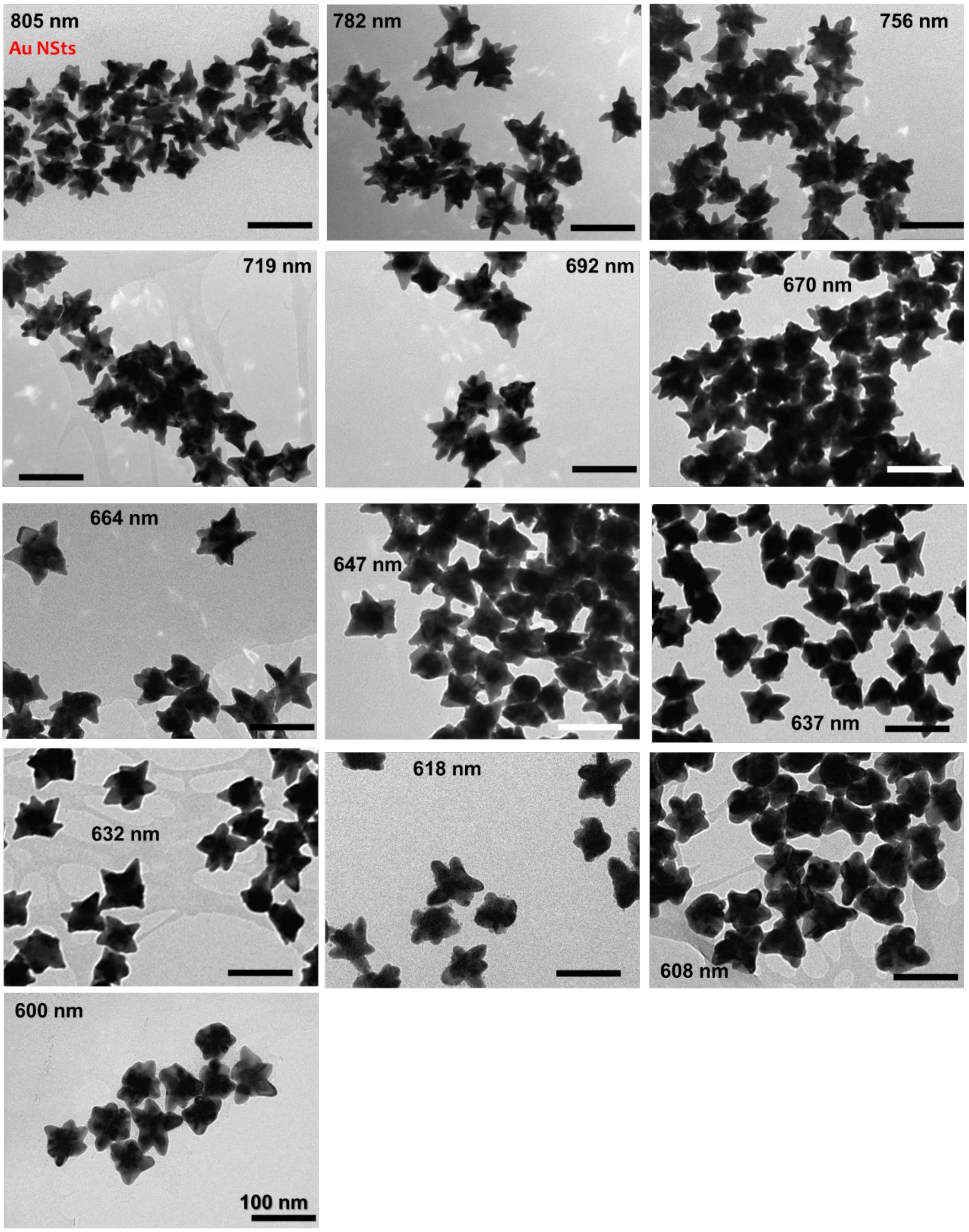

Figure S8. Representative TEM images of AuNSts (λ_{max} = 805 nm) and AuNSt@Ag particles (λ_{max} from 782 to 600 nm). Scale bars = 100 nm.

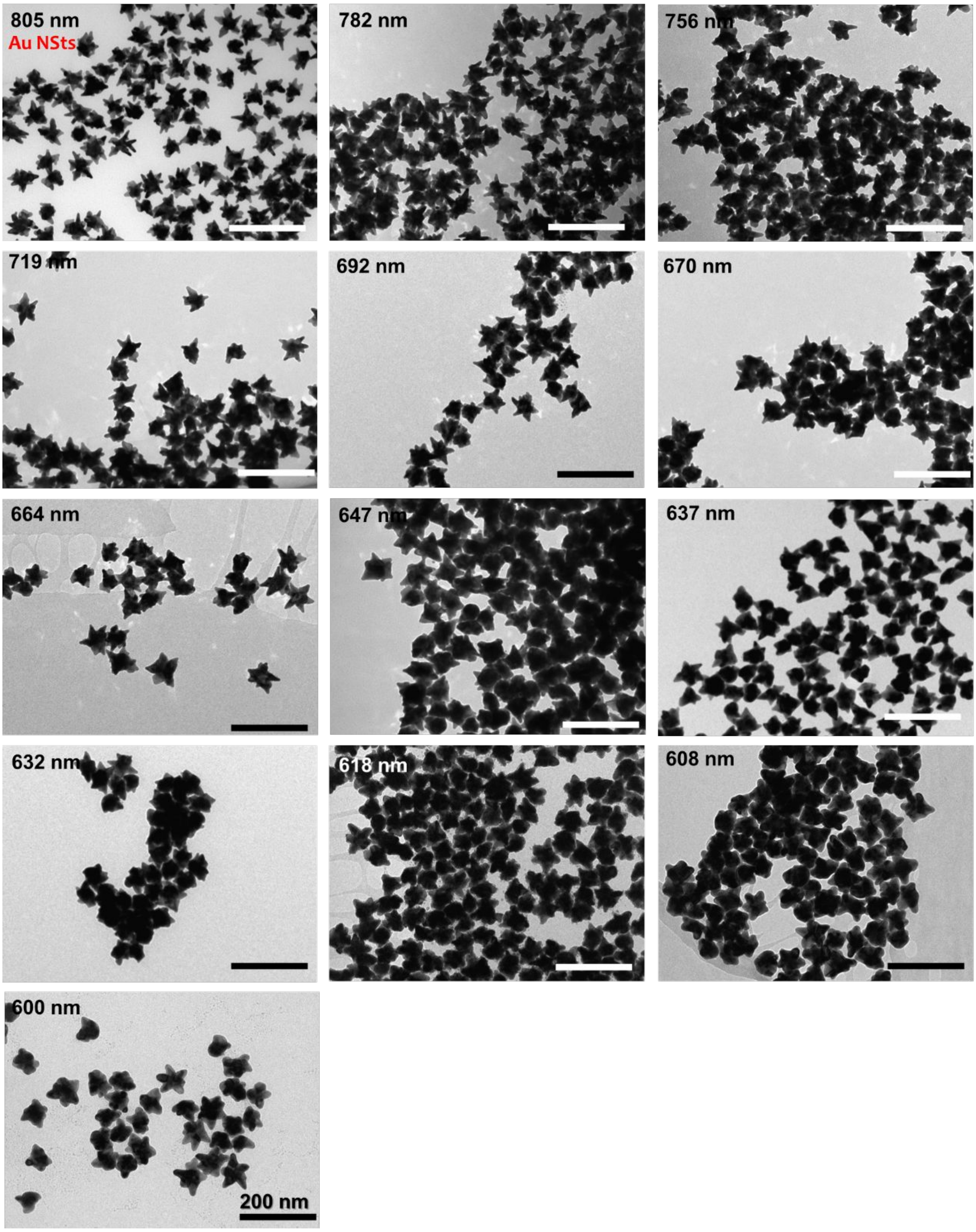

Figure S9. Representative TEM images of AuNSts (λ_{max} = 805 nm) and AuNSt@Ag particles (λ_{max} from 782 to 600 nm). Scale bars = 200 nm.

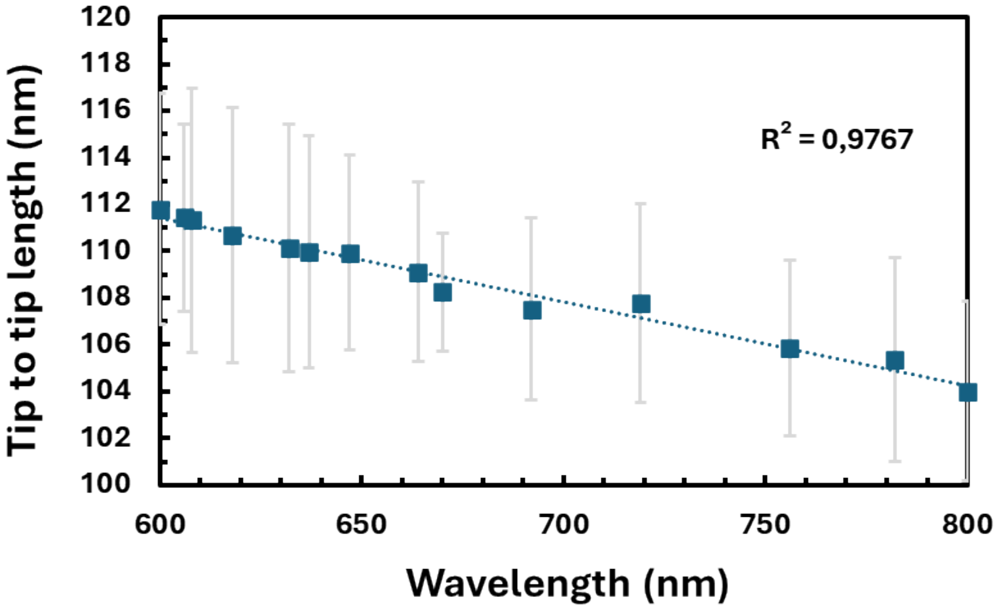

Figure S10. Plot showing the how the variation of the tip-to-tip length increases as the Ag is deposited. From ~104 nm for the AuNSt up to ~112 nm of the AuNSt@Ag with higher Ag content.

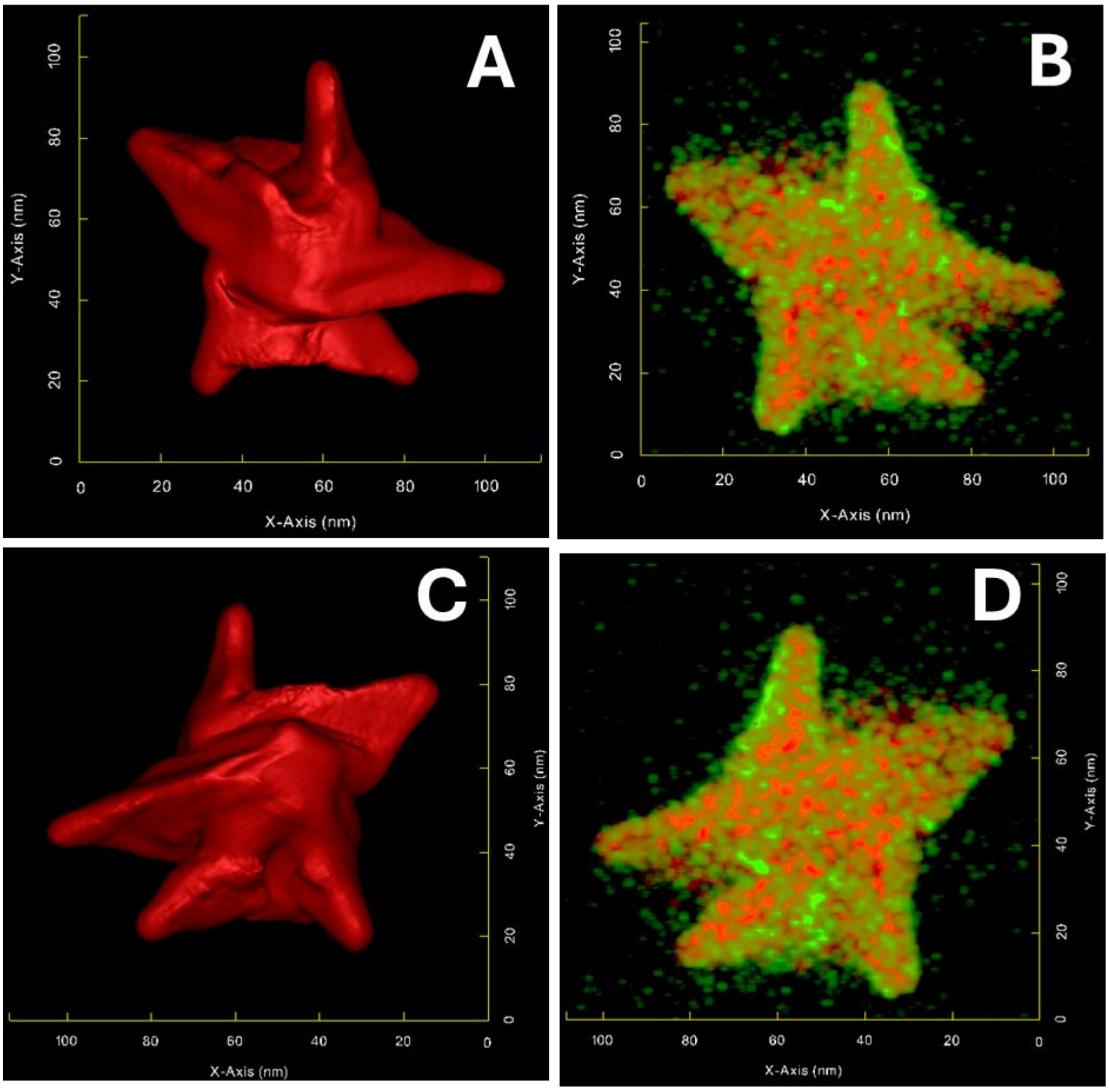

Figure S11. Electron tomography reconstruction images showing Z-axis projections of the synthesized AuNSt@Ag nanoparticles (λ_{max} = 620 nm) at two orientations (0˚ and 180˚). (A,C) HAADF-STEM images; and (B,D) corresponding EDX maps (Au depicted in red and Ag in green).

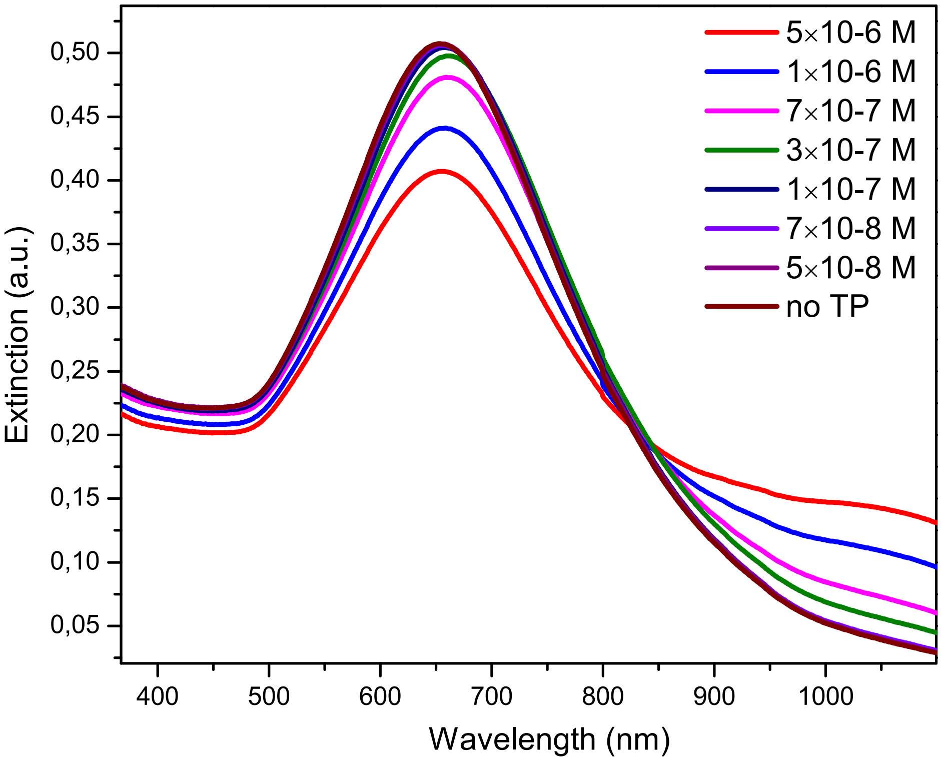

Figure S12. Extinction spectra of the TP-functionalized AuNSt@Ag colloids at different TP concentrations (after 2 hours of incubation).

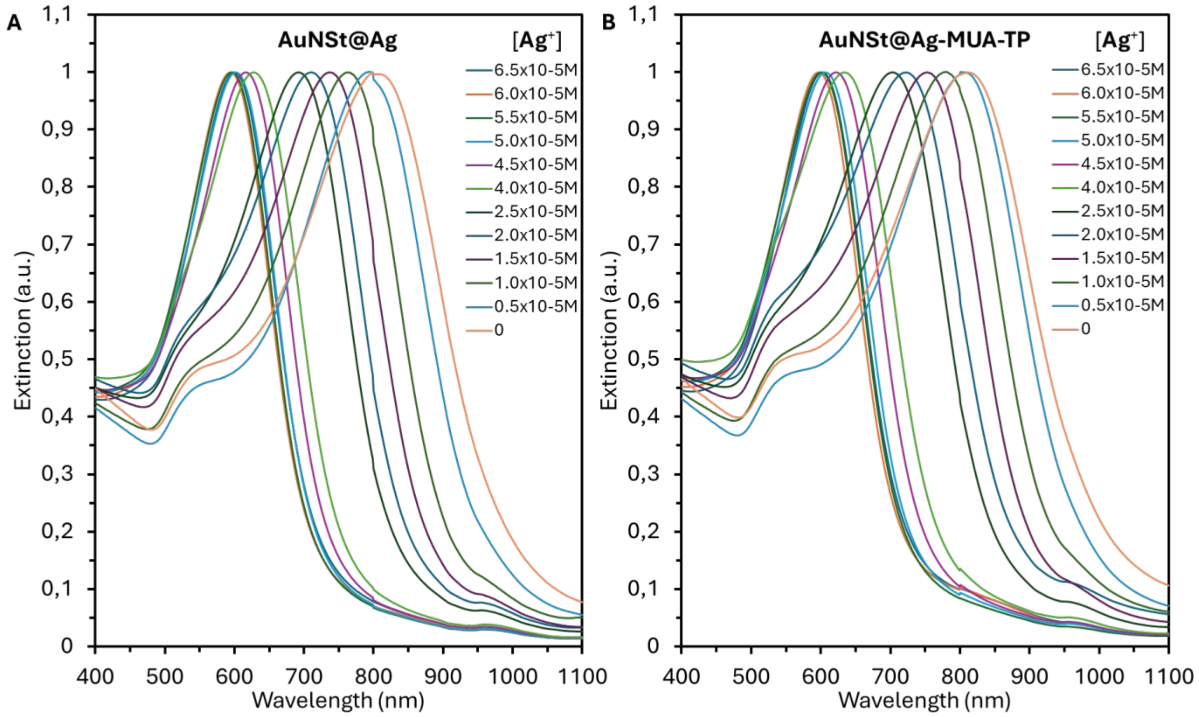

Figure S13. Extinction spectra of (A) the as-prepared AuNSt@Ag colloids and (B) the same colloids after functionalization with a mixed layer of MUA and TP, following 2 hours of incubation with each molecule.

ESTIMATION OF THE MUA+TP SURFACE COVERAGE IN THE SUBMONOLAYER REGIME

The concentration of spherical gold seed nanoparticles in the initial seed suspension ([Au^0] = 18.7 × 10^{-4} M), consisting of ~12%nm diameter particles, was estimated to be approximately 2.1 × 10^{13} NP/mL, based on the density of bulk gold (ρ = 19.3%g/cm³). From this seed suspension, 0.26 mL were used to prepare 35 mL of Au NSt, yielding a final [Au^0] of 2.8 × 10^{-4} M and an estimated number of total particles of 5.5 × 10^{12} NP/mL. To minimize particle loss during multiple centrifugation and washing steps, the number of mols of Au^0 per NSt was first estimated to be 1.8 × 10^{-18}. Since the final Au^0 concentration of the AuNSt suspension after the last centrifugation cycle was adjusted to 7.2 × 10^{-4} M, the corresponding number of nanostars in suspension was estimated to be 4.0 × 10^{11} NP/mL. For the preparation of the SERS samples, 0.18 mL of the Au NSt suspension were diluted to a final volume of 2 mL. Therefore, assuming minimal loss of the initial spherical seeds during the overall synthetic procedure, the final concentration of nanostars in the SERS-analyzed samples (both AuNSt@Ag and monometallic AuNSt) was estimated to be around 3.6 × 10^{13} NP/L.

Considering that the final concentrations of the surface ligands were MUA = 1.0 × 10^{-7} M and TP = 1.0 × 10^{-6} M for a total MUA+TP concentration of 1.1 × 10^{-6} M (i.e., 6.6 × 10^{17} molecules/L), and that both molecules occupy approximately 0.20–0.22 nm^2/molecule in self-assembled monolayers on gold surfaces,[1,2] the total metal surface required to accommodate all ligands would amount to approximately 1.5 × 10^{17} nm^2/L.

The irregular morphology of nanostars makes extremely difficult to experimentally quantify the overall surface area or to determine the number of surface-adsorbed TP molecules per particle. Therefore, we approached this issue from a conservative theoretical point of view, approximating the nanoparticles as 40%nm spheres, a geometry that provides a lower estimated surface area, as it is consistently smaller than that of any nanostar morphology tested in this study, as clearly visible in the TEM images. Based on our estimate of 3.6 × 10^{13} NP/L, the total metal surface available would be approximately 1.8 × 10^{17} nm^2/L. Thus, under these conditions, the combined MUA + TP

functionalization was designed to operate below monolayer coverage, irrespective of the specific morphology of the nanostructures.

This strategy avoided using large excesses of TP that could have skewed the results in favor of nanostructures with larger surface areas, as the resulting SERS intensity would have also reflected the total number of scatterers rather than solely their optical efficiency. Conversely, our approach ensured that all SERS measurements were performed on samples containing the same number of nanoparticles and the same number of TP molecules within the illuminated volume, under identical acquisition settings.

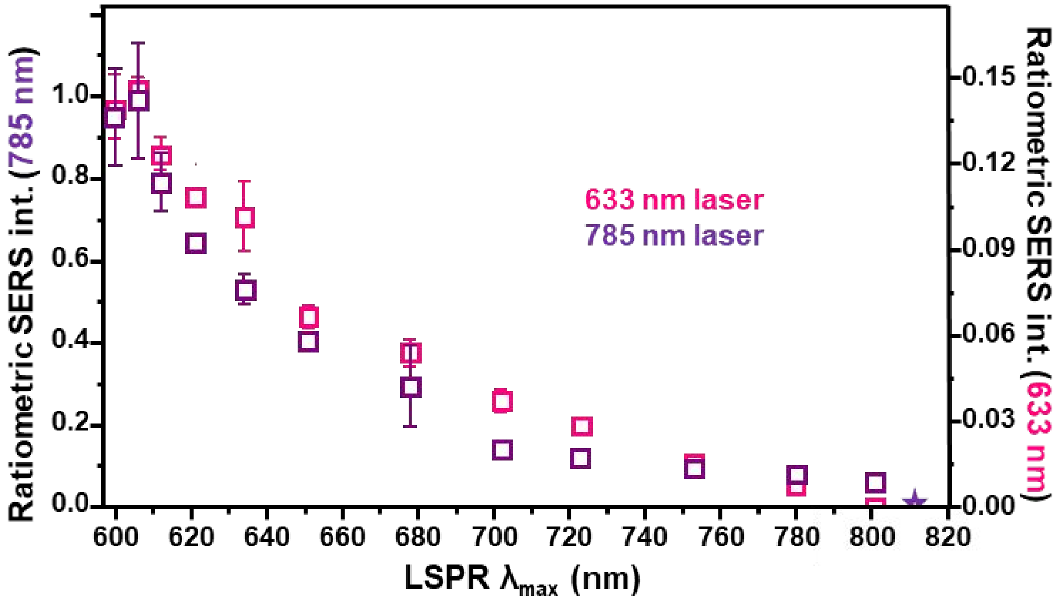

Figure S14. Ratiometric SERS intensity (I_{1570}/I_{1454}) plotted as a function of AuNSt@Ag LSPR λ_{max} for both 633 and 785 nm excitation lines. AuNSt@Ag nanoparticles were functionalized with a mixed MUA/TP layer.

REFERENCES

(1) Bain, C. D.; Troughton, E. B.; Tao, Y. T.; Evall, J.; Whitesides, G. M.; Nuzzo, R. G. Formation of Monolayer Films by the Spontaneous Assembly of Organic Thiols from Solution onto Gold. *J Am Chem Soc* 1989, *111* (1), 321–335. https://doi.org/10.1021/ja00183a049.

(2) Love, J. C.; Estroff, L. A.; Kriebel, J. K.; Nuzzo, R. G.; Whitesides, G. M. Self-Assembled Monolayers of Thiolates on Metals as a Form of Nanotechnology. *Chem. Rev.* 2005, *105* (4), 1103–1170. https://doi.org/10.1021/cr0300789.
